# Evidence for a Putative Isoprene Reductase in Acetobacterium wieringae

**DOI:** 10.1128/msystems.00119-23

**Published:** 2023-03-21

**Authors:** Miriam Kronen, Xabier Vázquez-Campos, Marc R. Wilkins, Matthew Lee, Michael J. Manefield

**Affiliations:** a UNSW Water Research Centre, School of Civil and Environmental Engineering, University of New South Wales, Sydney, NSW, Australia; b School of Biotechnology and Biomolecular Sciences, University of New South Wales, Sydney, NSW, Australia; Monash University

**Keywords:** isoprene reduction, isoprene fate, alternative electron acceptor, anaerobic respiration, methylbutene, putative isoprene reductase, *Acetobacterium* pangenome, putative isoprene-regulated operon

## Abstract

Recent discoveries of isoprene-metabolizing microorganisms suggest they might play an important role in the global isoprene budget. Under anoxic conditions, isoprene can be used as an electron acceptor and is reduced to methylbutene. This study describes the proteogenomic profiling of an isoprene-reducing bacterial culture to identify organisms and genes responsible for the isoprene hydrogenation reaction. A metagenome-assembled genome (MAG) of the most abundant (89% relative abundance) lineage in the enrichment, Acetobacterium wieringae, was obtained. Comparative proteogenomics and reverse transcription-PCR (RT-PCR) identified a putative five-gene operon from the A. wieringae MAG upregulated during isoprene reduction. The operon encodes a putative oxidoreductase, three pleiotropic nickel chaperones (2 × HypA, HypB), and one 4Fe-4S ferredoxin. The oxidoreductase is proposed as the putative isoprene reductase with a binding site for NADH, flavin adenine dinucleotide (FAD), two pairs of canonical [4Fe-4S] clusters, and a putative iron-sulfur cluster site in a Cys_6_-bonding environment. Well-studied *Acetobacterium* strains, such as A. woodii DSM 1030, A. wieringae DSM 1911, or A. malicum DSM 4132, do not encode the isoprene-regulated operon but encode, like many other bacteria, a homolog of the putative isoprene reductase (~47 to 49% amino acid sequence identity). Uncharacterized homologs of the putative isoprene reductase are observed across the *Firmicutes*, *Spirochaetes*, *Tenericutes*, *Actinobacteria*, *Chloroflexi*, *Bacteroidetes*, and *Proteobacteria*, suggesting the ability of biohydrogenation of unfunctionalized conjugated doubled bonds in other unsaturated hydrocarbons.

**IMPORTANCE** Isoprene was recently shown to act as an electron acceptor for a homoacetogenic bacterium. The focus of this study is the molecular basis for isoprene reduction. By comparing a genome from our isoprene-reducing enrichment culture, dominated by Acetobacterium wieringae, with genomes of other *Acetobacterium* lineages that do not reduce isoprene, we shortlisted candidate genes for isoprene reduction. Using comparative proteogenomics and reverse transcription-PCR we have identified a putative five-gene operon encoding an oxidoreductase referred to as putative isoprene reductase.

## INTRODUCTION

Isoprene represents the most abundant biogenic volatile organic compound (BVOC) on Earth and accounts for 70% of total annual BVOC emissions excluding methane (1–5). Large quantities of isoprene (500 to 600 Tg per year) (5–7) enter the atmosphere, making it an important participant in atmospheric chemistry, contributing to ozone and secondary organic aerosol (SOA) formation in the troposphere and increasing the lifetime of methane indirectly (8–13). Isoprene is mainly produced by plants but also by other organisms, such as Gram-positive and Gram-negative bacteria (14–18), fungi (19), and algae (20, 21).

Soils and marine environments harboring aerobic isoprene-degrading organisms serve as isoprene sinks (4, 22). The fate of isoprene under anoxic conditions was examined previously by our group (23), whereby isoprene was found to act as a 2e^−^ acceptor, with one C = C bond being reduced by an anaerobic enrichment culture to predominately 2-methyl-1-butene. Sequencing of 16S rRNA gene amplicons from this culture revealed enrichment of *Acetobacterium* to 92 to 100% relative abundance, with *Comamonadaceae* accounting for the rest (2 to 8%). The homoacetogenic *Acetobacterium* lineage dominating the H_2_-fed enrichment used 1.6 μmol isoprene h^−1^ as an electron acceptor in addition to HCO_3_^−^. Growth of the homoacetogen with isoprene produces 40% less acetate than with H_2_ and HCO_3_^−^ alone, suggesting that its reduction to methylbutene is coupled to energy conservation (23). More recently, these results were further validated by an independent study with a pure culture of *Acetobacterium* named “strain Y” (24). Strain Y also transformed isoprene to predominately 2-methyl-1-butene with a similar rate of 1.74 μmol isoprene h^−1^ (262.3 ± 21.2 μM day^−1^). However, Jin *et al.* suggest that isoprene hydrogenation in strain Y is a cometabolic process and is not linked to energy conservation. Besides isoprene, strain Y was also found to reduce 1,3-butadiene to 1-butene. Homoacetogens are known to reduce electron acceptors other than CO_2_, such as fumarate (25), nitrate (26), chloroethenes and chloroethanes (27), brominated aromatics (28), and acrylate derivatives (29). For instance, reduction of the functionalized C = C bond (C = C bond conjugated to an electron-withdrawing group) in caffeate by the model organism Acetobacterium woodii (29–31) is a well-studied example for CO_2_-alternative electron acceptors in *Acetobacterium* species. Interestingly, pure cultures of A. woodii DSM 1030, A. malicum DSM 4132, and A. wieringae DSM 1911 showed no isoprene-reducing activity (23), suggesting that the isoprene hydrogenation capability is not a mutual trait among all *Acetobacterium* spp.

This study aimed to identify microorganisms and their corresponding genes/enzymes involved in the reduction of the unfunctionalized C = C bond in isoprene. DNA from an isoprene-reducing enrichment culture was sequenced, and protein profiles with and without isoprene were compared. Metagenomic and comparative metaproteomic analyses implicate a putative oxidoreductase in isoprene reduction encoded in a putative five-gene operon.

## RESULTS

### A putative oxidoreductase encoded by Acetobacterium wieringae is upregulated by isoprene.

Cell suspension experiments with the *Acetobacterium*-dominated (relative abundance 16S rRNA gene amplicon sequencing of 92 to 100%) enrichment culture (23) pregrown on H_2_/HCO_3_^−^ indicated that isoprene reduction is induced in the presence of isoprene, H_2_, and HCO_3_^−^ (Fig. S1 in the supplemental material). Therefore, label-free comparative metaproteomics was performed to identify proteins and corresponding genes involved in isoprene metabolism. To generate a database for protein identification, the isoprene-reducing enrichment culture was grown on H_2_/HCO_3_^−^/±isoprene, and the extracted DNA was sequenced. Over 7.5 million paired-end reads were used to assemble 338 contigs. Two nearly complete metagenome-assembled genomes (MAGs) were obtained (Table 1). No other lineages were detected. MAG ISORED-1 showed 74% average amino acid identity (AAI) and 79% average nucleotide identity (ANI) to Comamonas aquatica CJD (see Table S1 at https://doi.org/10.6084/m9.figshare.22012931), and ISORED-2 showed 97% AAI and ANI to Acetobacterium wieringae DSM 1911 (see Table S2 at https://doi.org/10.6084/m9.figshare.22012931). MAG ISORED-2 is dominant in the enrichment based on relative abundance (77%) and more so when cultivated in the presence of isoprene (relative abundance of 88.71%) (Table 1).

**TABLE 1:** Summary of metagenome assembled genomes from the isoprene reducing cultures

MAGs	Genome size (mbp)	GC content (%)	Completeness/redundancy (%)	Mean coverage	Relative abundance (% metagenome)		Relative abundance (% biomass)^*a*^	
					H_2_/HCO_3_^−^	Isoprene	H_2_/HCO_3_^−^	Isoprene
*Comamonas* sp. ISORED-1 (GCA_902175065.1)	3.22	63.7	98.28–99.3/0.72–3.45	50.8×	22.99	11.29	15.54	6.41
Acetobacterium wieringae ISORED-2 (GCA_902175055.1)	3.81	44	92.93–99.28/0.95–5.76	244.3×	77.01	88.71	84.46	93.59

a Biomass contribution was calculated according to (131).

10.1128/msystems.00119-23.2 FIG S1 Induction of isoprene reduction during acetogenesis from H_2_ plus CO_2_ by isoprene-reducing culture dominated by A. wieringae. (A and B) Cell suspensions of enrichment culture pregrown on H_2_/HCO_3_^−^ without (A) and with (B) isoprene were incubated with shaking under N_2_ atmosphere at 30°C in the presence of 5 × 10^4^ Pa H_2_, 40 mM HCO_3_^−^, and 1 mM isoprene. Note that the time scales between A and B are different, and that on the left *y* axis the units are nmols per microcosm and on the right *y* axis the units are μmol per microcosm. Note that these data are from a single, representative experiment but have been repeated at least twice. Download FIG S1, PNG file, 0.4 MB. Copyright © 2023 Kronen et al. 2023 Kronen et al. https://creativecommons.org/licenses/by/4.0/ This content is distributed under the terms of the Creative Commons Attribution 4.0 International license.

**FIG 1:**
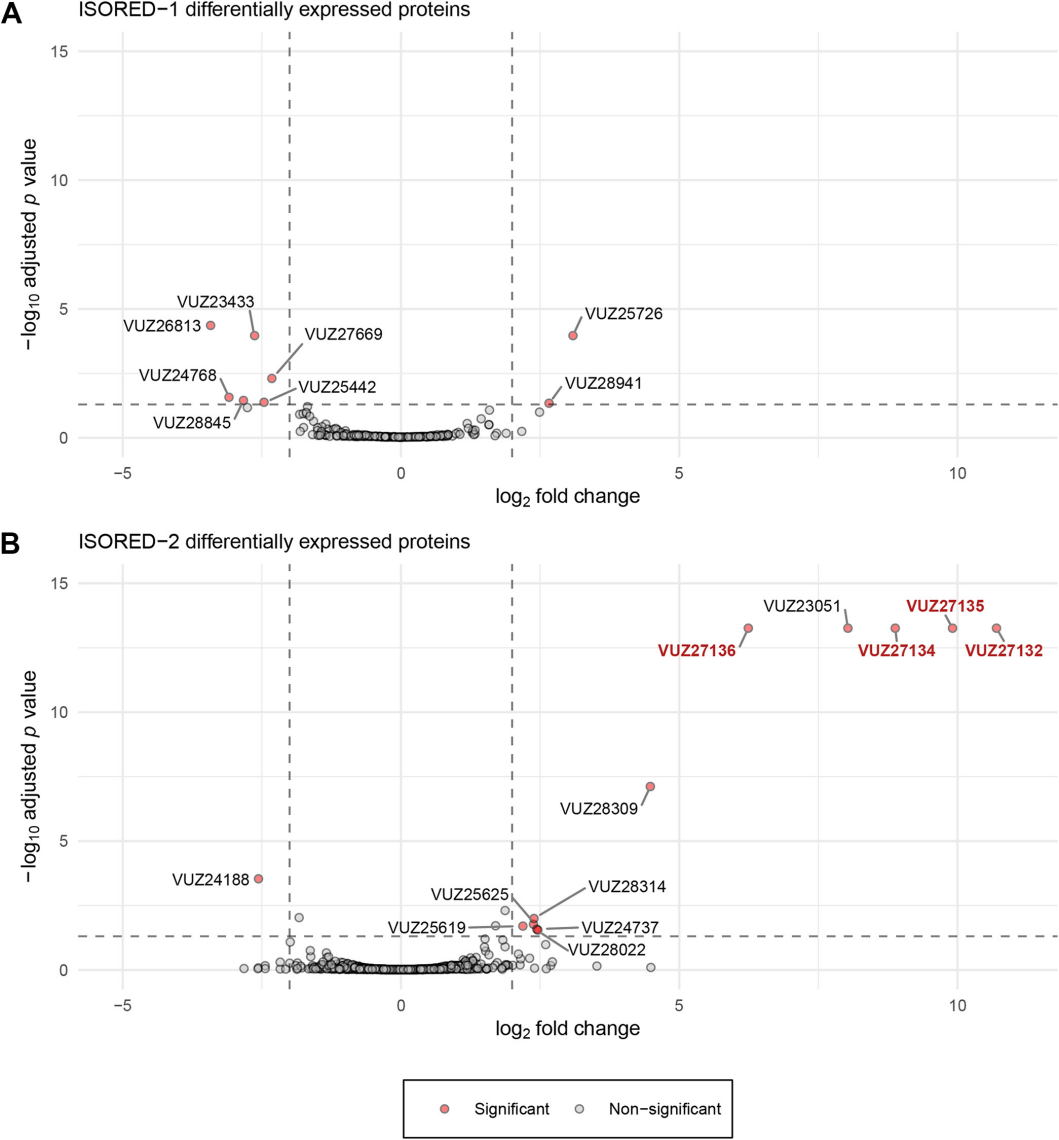
Volcano plot of the metaproteomic data comparing cells grown on H_2_/HCO_3_^−^/isoprene versus H_2_/HCO_3_^−^. Significant data points (colored) are based on a LFC of ±2 and an adjusted *P* value of ≤0.05. Labeling of the significant points is based on metagenome-assembled genomes ISORED-1 (A; *Comamonas* sp.) and ISORED-2 (B; Acetobacterium wieringae). Proteins located adjacent to each other in the genome of *A. wieringae* ISORED-2 (B) are highlighted. Data were obtained from 4 replicates for each growth condition (PRIDE database PXD023683). Differential expression analysis was conducted with limma (130).

Differential metaproteomes were generated by liquid chromatography-tandem mass spectrometry (LC-MS/MS) analysis of proteins obtained from cultures grown on H_2_/HCO_3_^−^ with and without isoprene. A total of 1,531 proteins were identified. Consistent with the dominance of the homoacetogen in culture, 1,279 proteins (83.5%) belonged to A. wieringae ISORED-2 (Fig. 1B), and 252 proteins (16.5%) were assigned to *Comamonas* ISORED-1 (Fig. 1A). This is also reflected in the estimated biomass contributions, where A. wieringae ISORED-2 constituted ~94% and *Comamonas* ISORED-1 constituted ~6% relative abundance of biomass of the community (Table 1).

Differential expression analysis identified significant changes (false-discovery rate [FDR] of ≤0.05; absolute value of log_2_ fold change [LFC] ≥ 2) in 12 proteins from A. wieringae ISORED-2 and 8 proteins from *Comamonas* sp. ISORED-1 between cells grown on H_2_/HCO_3_^−^/isoprene and those grown on H_2_/HCO_3_^−^ (Table 2). Only 13 proteins were significantly more abundant in isoprene-exposed cells, 11 belonging to A. wieringae ISORED-2 and 2 belonging to *Comamonas* sp. ISORED-1 (Fig. 1A and B; Table 2).

**TABLE 2:** List of proteins that significantly differed in abundance between cells grown on H_2_/HCO_3_^−^/isoprene and cells grown on H_2_/HCO_3_^−^

MAG^*a*^	Locus tag	Accession no.	LFC^*b*^	*P* value (adj)^*b*^	Size amino acids	EggNOG^*c*^	Protein prediction	Function^*f*^
ISORED-1	ISORED1_01953	VUZ26813.1	−3.42	4.35 × 10^−5^	240	ENOG4105VNB	Acyl carrier protein, AcpP	Synthesis of fatty acids
	ISORED1_01103	VUZ24768.1	−3.09	2.67 × 10^−2^	1,314	ENOG4105DEA	Trigger factor	Ribosome-associated chaperone
	ISORED1_00277	VUZ28845.1	−2.83	3.54 × 10^−2^	534	ENOG4108WFY	Phasin	Accumulation of polyhydroxyalkanoates
	ISORED1_00539	VUZ23433.1	−2.63	1.08 × 10^−4^	1,248	ENOG4105C65	Serine hydroxymethyltransferase, glyA	Interconversion of serine and glycine with THF^*d*^ serving as the one-carbon carrier
	ISORED1_01462	VUZ25442.1	−2.46	4.17 × 10^−2^	867	ENOG4105J80	ATPase gamma chain	Regulates ATPase activity and flow of protons through the CF_0_^*e*^ complex
	ISORED1_02337	VUZ27669.1	−2.32	4.97 × 10^−3^	213	ENOG4105VCC	30S ribosomal protein S21	Structural component of the ribosome. Binds rRNA.
	ISORED1_02798	VUZ28941.1	2.66	4.55 × 10^−2^	1,272	ENOG4105CHM	Aminotransferase, AlaA	Pyridoxal-dependent aminotransferase
	ISORED1_01561	VUZ25726.1	3.09	1.08 × 10^−4^	2,256	ENOG4105CWR	ppGpp synthetase/hydrolase, SpoT	Guanosine-3′,5′-bis(diphosphate) 3′-pyrophosphohydrolase
ISORED-2	ISORED2_02175	VUZ24188.1	−2.56	2.91 × 10^−4^	281	ENOG4108S8M	Degv family	Unknown
	ISORED2_02836	VUZ25619.1	2.19	1.99 × 10^−3^	461	ENOG4105H0F	Uroporphyrinogen decarboxylase (URO-D)	Porphyrin-containing compound metabolic process
	ISORED2_02841	VUZ25625.1	2.38	1.69 × 10^−2^	149	ENOG41083D8	Heat shock protein	Stress response
	ISORED2_01001	VUZ28314.1	2.39	1.01 × 10^−2^	556	ENOG4105CKU	Formyltetrahydrofolate synthetase	One-carbon metabolic process
	ISORED2_02500	VUZ24737.1	2.44	2.62 × 10^−2^	67	ENOG4105VJV	50s ribosomal protein L35	Structural constituent of ribosome
	ISORED2_00772	VUZ28022.1	2.46	2.85 × 10^−2^	513	ENOG410ND7V	Xylulokinase	Carbohydrate metabolic process
	ISORED2_00996	VUZ28309.1	4.48	7.63 × 10^−8^	167	ENOG4105WDP	Acetyltransferase	*N*-Acetyltransferase activity
	**ISORED2_03549**	**VUZ27136.1**	**6.24**	**5.48 × 10^−14^**	**117**	**ENOG4105WMM**	**Hydrogenase accessory protein HypA**	**Plays a role in a hydrogenase nickel cofactor insertion step**
	ISORED2_01586	VUZ23051.1	8.03	5.48 × 10^−14^	327	ENOG41060FG	Methyltransferase 1 (EC 2.1.1.-)	Unknown
	**ISORED2_03547**	**VUZ27134.1**	**8.88**	**5.48 × 10^−14^**	**225**	**ENOG4107RSS**	**Hydrogenase accessory protein HypB**	**Nickel cation binding, transition metal ion binding hydrolase activity**
	**ISORED2_03548**	**VUZ27135.1**	**9.91**	**5.48 × 10^−14^**	**350**	**ENOG4105DQ9**	**4Fe-4S ferredoxin**	**Iron-sulfur cluster binding and electron carrier activity**
	**ISORED2_03545**	**VUZ27132.1**	**10.70**	**5.48 × 10^−14^**	**901**	**ENOG4107QZ5**	**Glutamate synthase, FAD-dependent oxidoreductase**	**Oxidoreductase activity; iron-sulfur cluster binding; flavin adenine dinucleotide binding oxidoreductase activity**

a Proteins encoded by genes located adjacent to each other are highlighted in bold and gray. Data were obtained from 4 replicates for each growth condition (PRIDE database PXD023683).

b Significant data points are based on a minimum absolute value LFC of 2 and an adjusted *P* value of 0.05.

c EggNOG matches are shown with their functional description.

d THF, tetrahydrofolate.

e CF_0_, chloroplast ATP synthase.

f Function is based on the EggNOG orthologous.

**FIG 2:**
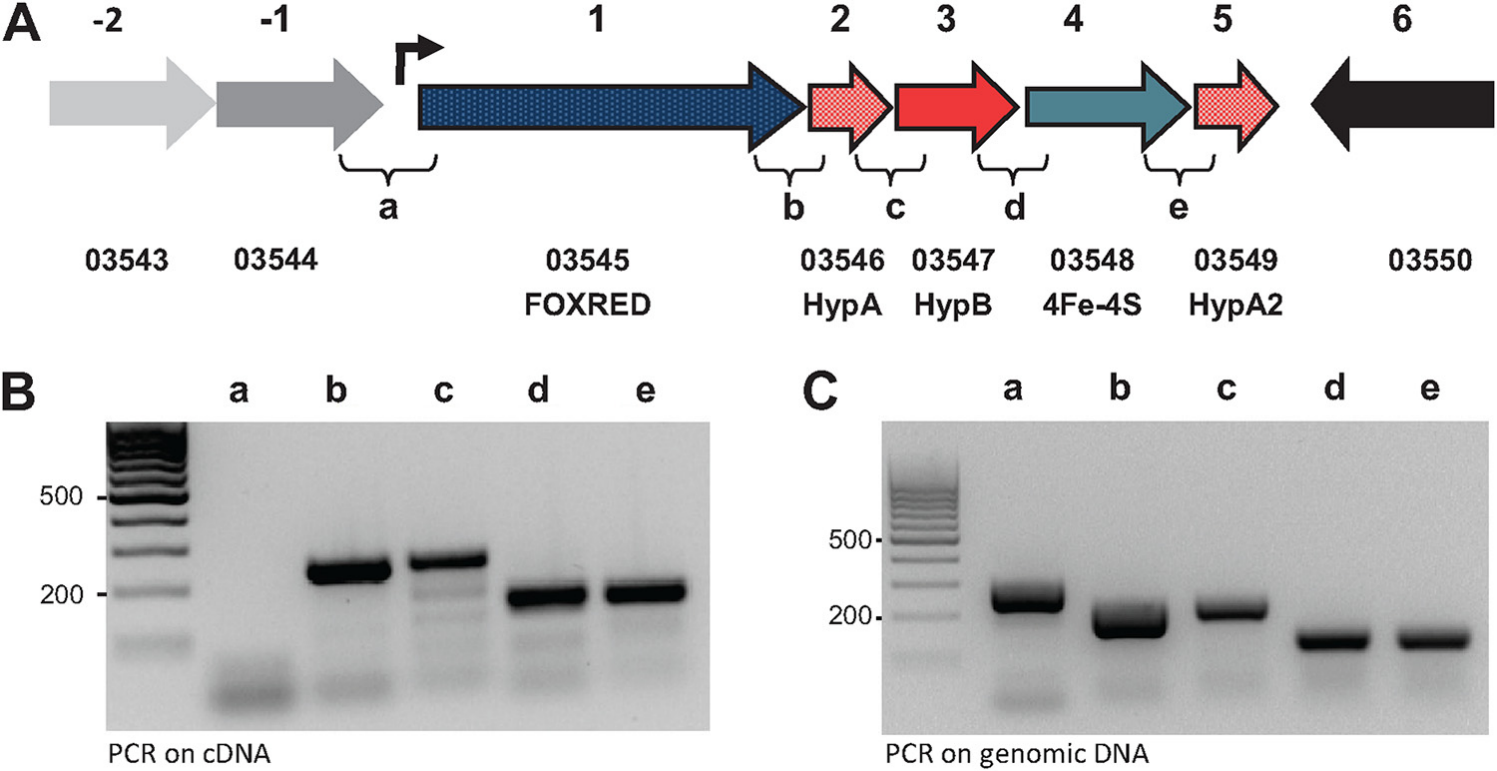
Organization of genes upregulated by isoprene and confirmation of the operon structure by RT-PCR. (A) The five-gene operon harbors genes encoding a putative FAD-dependent oxidoreductase (FoxRed, ISORED2_03545, blue), three nickel-inserting hydrogenase maturation factors (two HypA [ISORED2_03546 and ISORED2_03549, light red] and one HypB [ISORED2_03547, dark red]), and one 4Fe-4S ferredoxin (ISORED2_03548, turquoise); diagrammatic representation of the operon with open reading frames (ORF) 1 to 5 and their intersections (a to e) are annotated. (B) Amplicons with primers connecting intersections of the neighboring ORFs on the cDNA transcript. (C) Amplicons with primers connecting intersections of the neighboring ORFs on chromosomal DNA positive control. No bands appeared in negative controls lacking reverse transcriptase.

Five out of 12 isoprene-responsive proteins from A. wieringae were more significantly abundant (FDR ≤ 0.05), with a LFC of 6.2 to 10.7, than the remaining 6 (LFC of 2.5 to 4.8), and 1 protein was less expressed (VUZ24188.1, LFC of −2.56). Four (VUZ27132.1, VUZ27134.1, VUZ27135.1, and VUZ27136.1) out of these five are adjacent to each other in the ISORED-2 MAG, indicating that they might belong to the same operon (Fig. 1B and 2A; Table 2). Protein VUZ27132.1 (Table 2) is predicted to be a molybdopterin oxidoreductase, with the best orthologous group match ENOG4107QZ5. Protein VUZ27136.1 (ENOG4105WMM) is a HypA homolog, and VUZ27134.1 (ENOG4107RSS) is a HypB homolog. HypA and HypB proteins are typically responsible for the acquisition and insertion of nickel in the catalytic center of [NiFe]-hydrogenases; it should be noted here that the genome of A. wieringae ISORED-2 does not encode the structural genes for a [NiFe]-hydrogenase. Protein VUZ27135.1 (ENOG4105DQ9) belongs to the 4Fe-4S ferredoxin superfamily (SSF54862), but the sequence is not affiliated with any specific family. Protein VUZ23051.1 (ENOG41060FG, methyltransferase) is also highly expressed (LFC of 8.03) but is not part of the operon. Predicted protein functions from the six less isoprene-responsive proteins (LFC of 2.5 to 4.8) include acetyltransferase, xylulokinase, uroporphyrinogen decarboxylase, heat shock protein, formyltetrahydrofolate synthetase, and 50S ribosomal protein L35 (Table 2).

Only two proteins in *Comamonas* sp. ISORED-1 were significantly more abundant (FDR ≤ 0.05) following isoprene exposure: protein VUZ25726.1 (ENOG4105CWR, LFC of 3.09), a (p)ppGpp synthetase/hydrolase (SpoT), and protein VUZ28941.1 (ENOG4105CHM, LFC of 2.66), an aminotransferase (AlaA) (Table 2). SpoT is typically involved in the stringent response (32), a ubiquitous stress signaling pathway that enables bacteria to respond to nutrient starvation, and AlaA catalyzes the reversible transamination reaction pyruvate + glutamate ↔ l-alanine + α-ketoglutaric acid. *Comamonas* sp. ISORED-1 had 6 proteins that were significantly less abundant (LFC of −3.42 to −2.31) following isoprene exposure: an acyl carrier protein (AcpP), a trigger factor (ribosome-associated chaperon), phasin (involved in the accumulation of polyhydroxyalkanoates), serine hydroxymethyltransferase (glyA), ATPase gamma chain, and 30S ribosomal protein S21 (Table 2).

### The putative isoprene operon.

Apart from A. wieringae ISORED-2’s oxidoreductase (VUZ27132.1), no isoprene-responsive protein from MAG ISORED-2 or ISORED-1 is predicted by protein function to be involved in redox processes (Table 2). This makes the oxidoreductase from the A. wieringae lineage the only likely candidate that could catalyze the isoprene hydrogenation reaction. The relevant gene of A. wieringae’s oxidoreductase (VUZ27132.1) is organized in one operon together with the genes of three other isoprene-induced proteins (VUZ27134.1, VUZ27135.1, and VUZ27136.1) and an additional gene (encoding a HypA protein, VUZ27133.1) not significantly more abundant in the metaproteome (Fig. 1A). All five genes have the same orientation, with intergenic regions ranging between 7 and 71 nucleotides. Operon prediction analysis (FGENESB) suggested that the five genes are transcribed as an operon (Fig. 2A; Fig. S2). Reverse transcription-PCR (RT-PCR) using primer sets flanking individual intergenic regions of adjacent genes (see Tables S3 and S4 at https://doi.org/10.6084/m9.figshare.22012931) also indicated that the genes are transcribed as an operon (Fig. 2B and C). For promoter prediction analysis (BPROM), see Text S1 and Fig. S2.

10.1128/msystems.00119-23.1 TEXT S1 Additional results regarding the induction of isoprene-reducing activity, prediction of promoters for the *isr* operon, and genome environment of the *isr* operon. Download Text S1, DOCX file, 0.03 MB. Copyright © 2023 Kronen et al. 2023 Kronen et al. https://creativecommons.org/licenses/by/4.0/ This content is distributed under the terms of the Creative Commons Attribution 4.0 International license.

10.1128/msystems.00119-23.3 FIG S2 Promoter region of the isoprene operon. Prediction analysis (BPROM) revealed a potential transcription start site around 44 bp upstream of the open reading frame (ORF; ISORED2_03545) start codon. Two potential transcription factor (TF; RNA polymerase sigma factor rpoD17 and Ihf) binding sites were predicted at 58 bp and 38 bp upstream of the ORF 1 start codon. Additionally, 4 TF binding sites could be identified 13 bp upstream of the ISORED2_03545 start codon. These are highly suggestive binding sites for a Fur (ferric uptake regulator) or NikR (nickel uptake regulator) type of TF. Download FIG S2, PNG file, 0.04 MB. Copyright © 2023 Kronen et al. 2023 Kronen et al. https://creativecommons.org/licenses/by/4.0/ This content is distributed under the terms of the Creative Commons Attribution 4.0 International license.

### Isoprene reduction and the putative isoprene-regulated operon is unique to ISORED-2 among known *Acetobacterium* species.

While A. wieringae DSM 1911 is the same species as A. wieringae ISORED-2, it did not catalyze isoprene reduction nor did A. woodii DSM 1030, A. malicum DSM 4132, or A. dehalogenans DSM 11527 (23). Comparative pangenome analysis with eight publicly available *Acetobacterium* genomes (see Table S5 at https://doi.org/10.6084/m9.figshare.22012931) was used to assess the distribution of the putative isoprene-regulated operon encoding the oxidoreductase (VUZ27132.1) within the *Acetobacterium* genus and to find features unique to the A. wieringae ISORED-2 MAG.

Pangenome analysis showed the protein-coding sequences from all nine genomes (33,035 in total) grouped into 8,190 gene clusters, based on an Markov Clustering Algorithm (MCL) inflation value of 6 (parameter controlling the granularity of the clustering) (Fig. S3). A shared set of 1,492 gene clusters (core) is shown across the nine genomes along with protein sets that are unique in each of the *Acetobacterium* genomes (Fig. S3 and Table S6 at https://doi.org/10.6084/m9.figshare.22012931).

10.1128/msystems.00119-23.4 FIG S3 Pangenome analysis of nine *Acetobacterium* genomes. A list of the complete *Acetobacterium* genomes used for pangenome analysis with anvi’o are available in Table S5 at https://doi.org/10.6084/m9.figshare.22012931. Each of the 8,190 gene clusters contains one or more genes contributed by one or more isolate genomes. The “core” selection corresponds to the gene clusters that contain genes from all the genomes. The “soft core” selection corresponds to gene clusters that contain genes from at least 7 genomes and the shell from at least 4 genomes. “Singletons” selection corresponds to clusters that contain one or multiple genes from a single genome. Genes unique to the A. wieringae ISORED-2 MAG and other *Acetobacterium* genomes or MAGs are shown (see Table S6 at https://doi.org/10.6084/m9.figshare.22012931). Inset bar graphs show the number of gene clusters, percent genome completion, percent redundancy, and total length for each lineage. Download FIG S3, PNG file, 0.4 MB. Copyright © 2023 Kronen et al. 2023 Kronen et al. https://creativecommons.org/licenses/by/4.0/ This content is distributed under the terms of the Creative Commons Attribution 4.0 International license.

For the A. wieringae ISORED-2 MAG, a total repertoire of 3,628 proteins in 3,386 gene clusters were identified. Of these gene clusters, 318 with 352 proteins were unique to the *A*. *wieringae* ISORED-2 MAG (see Table S7 at https://doi.org/10.6084/m9.figshare.22012931). Regardless of the MCL inflation value (2, 4, or 6) used, the oxidoreductase (VUZ27132.1) from the putative isoprene-regulated operon was always located in a singleton gene cluster. Among the analysed *Acetobacterium* genomes, four genes in the putative isoprene-regulated operon are unique to the ISORED-2 MAG: 4Fe-4S ferredoxin, two HypA proteins, and the oxidoreductase (VUZ27132.1). This is consistent with the absence of isoprene-reducing ability in A. woodii DSM 1030, A. wieringae DSM 1911, A. malicum DSM 4132, and A. dehalogenans DSM 11527.

**FIG 3:**
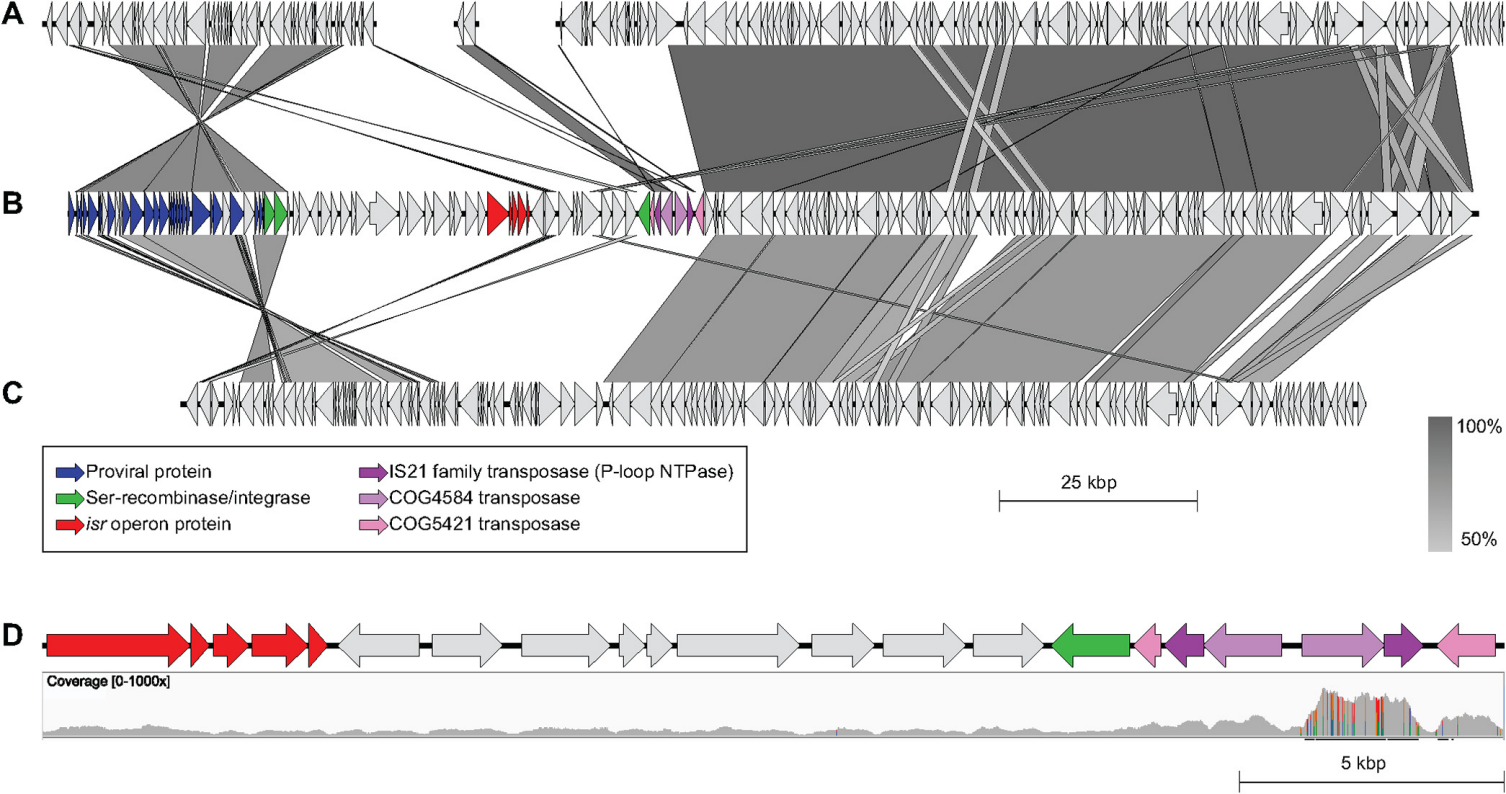
Details of the reassembled contig containing the putative isoprene-regulated operon. (A to C) Homology between A. wieringae DSM 1911 (A; contigs LKEU01000044, 59, and 37), A. wieringae ISORED-2 (B), and *A.* sp. KB-1 (C; between 925 and 1,075 kbp). The grayscale gradient indicates the percent identity (Easyfig BLASTn minimum size of 100, E value of 1 × 10^−5^, minimum identity of 50%). (D) Close view at the coordinates between the isoprene-regulated operon and the IS elements highlighting the coverage of the region (maximum of 748×).

The putative isoprene-regulated operon (here referred to as the *isr* operon) is located between 69,745 and 75,048 bp in a 90,374-bp contig (ISORED_48, Fig. 3B). The first half of this contig contains mainly protein-coding genes of viral origin. The mean contig coverage and the coverage of the proviral portion are close to the values for the MAG, indicating no active viral replication. The provirus (*Siphoviridae*) showed an average amino acid identity of 59.11% with the *Erysipelothrix* phage Φ1605 (33) based on CheckV, and tBLASTx of many of the viral proteins also showed similar identities with several Streptococcus phages (34). This proviral region also appears in other *Acetobacterium* spp. genomes, including A. wieringae DSM 1911 and *A.* sp. KB-1 (Fig. 3A and C). The contig contains three different Ser recombinases (integrases). Two of them adjacent to the provirus (Fig. 3B) show very high identity values with recombinases found in A. wieringae DSM 1911, *A.* sp. MES1, and *A.* sp. KB-1 and might be part of the provirus. Aside from the region of the terminal recombinases, contig ISORED_48 is nearly identical (3 bp difference) to the recently released sequence of Acetobacterium wieringae strain Y (GCA_025813735.1) (24). This high similarity is surprising considering this is a highly dynamic region due to the involvement of a prophage and a number of mobile genetic elements flanking the contig (e.g., recombinases and insertion sequences). This is also unexpected due to the geographically distant isolation sources.

As the provirus and recombinases were the main sequences sharing some degree of synteny in the original contig with assembled *Acetobacterium* spp. genomes, the contig encoding the putative *isr* operon was scrutinized in more detail and the MAG reassembled to better understand the gene environment of the operon (Text S1). The reassembled ISORED-2 MAG was ~40 kbp larger, with a more polished putative *isr* operon contig (Fig. 3B and D), which showed a higher degree of synteny with the genome of *Acetobacterium* sp. KB-1 (Fig. 3C). While the reassembly process was able to extend the putative *isr* operon contig, it did so at the expense of collapsing several insertion sequences (ISs). Most of the ISs in this region are suspected to appear in tandem repeats based on their higher apparent sequence coverage.

### Taxonomic distribution and phylogeny of isoprene-induced oxidoreductase homologs.

**FIG 4:**
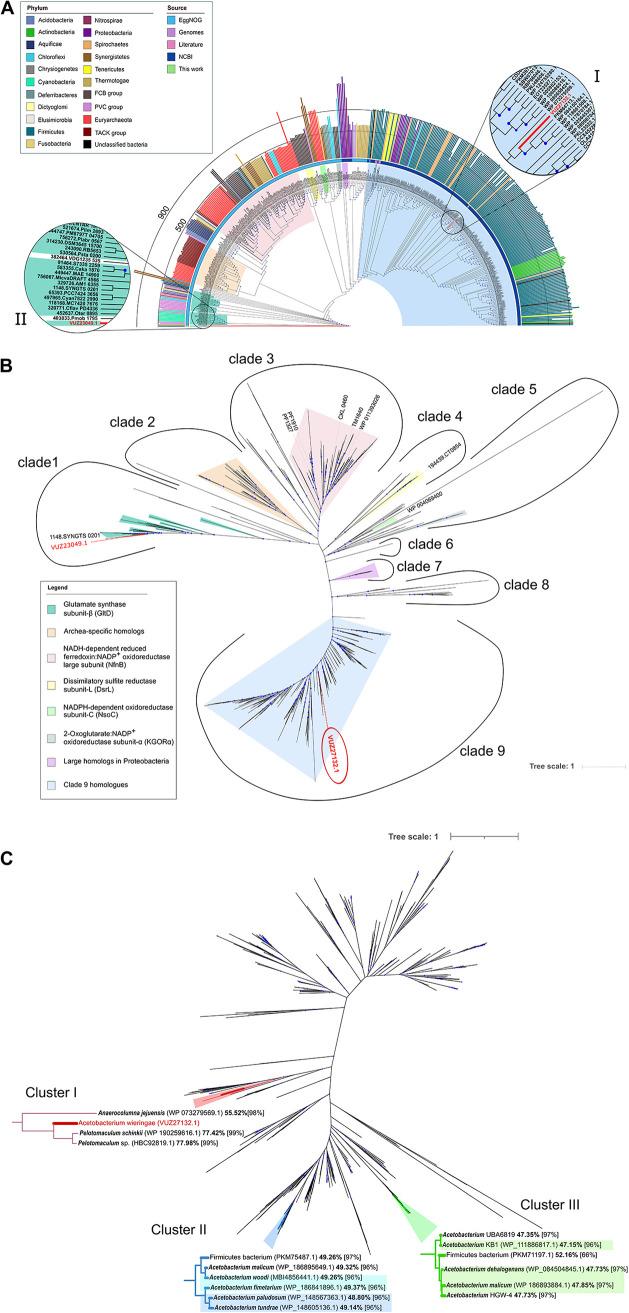
(A) Unrooted maximum likelihood phylogenetic tree of IsrA (VUZ27132.1) and its homologs. Phylogeny of amino acid sequences of IsrA and its homologs are drawn as an unrooted circular tree. Highlighted are the positions of protein VUZ27132.1 (I) and another VUZ27132.1 ortholog from A. wieringae ISORED-2 VUZ23049.1 (II). The outer circle shows bars representing the protein length colored according to phylum. The outer ring indicates the source of the sequences. Clades are colored according to the clades in the unrooted tree (B). (B) The phylogenetic analysis of IsrA and all its homologs revealed nine distinct clades containing redox enzymes involved in different reactions. Blue circles indicate an ultrafast bootstrap support of ≥90%. Proteins labeled in black indicate characterized proteins. See Table S7 at https://doi.org/10.6084/m9.figshare.22012931 for information about each sequence. See also the interactive iTol link https://itol.embl.de/tree/4918010318257311557452600. (C) Extended phylogenetic analysis of clade 9 homologs. Subtrees show clusters of IsrA homologs from *Acetobacterium* spp. and the closest homologs to IsrA (*Pelotomaculum* spp.). Blue circles indicate an ultrafast bootstrap support of ≥90%. Amino acid identity values are shown next to the name followed by sequence coverage between brackets. Highlighted *Acetobacterium* names indicate identical gene arrangement (refer to Fig. S4 in the supplemental material). See Table S8 for information about each sequence at https://doi.org/10.6084/m9.figshare.22012931.

The A. wieringae oxidoreductase (VUZ27132.1) was searched within the EggNOG and NCBI databases to determine its broader distribution and to find biochemically characterized homologs. Protein VUZ27132.1, which is 901 amino acids long, is predicted to belong to the bacterial orthologous group ENOG4107QZ5 (141 proteins, 99 species), with an archaeal counterpart in the orthologous group arCOG01292 (88 proteins, 55 species). The ENOG4107QZ5/arCOG01292 orthologous groups primarily (92%) contain proteins ~500 to 600 amino acids long, whereas the NCBI BLAST search primarily (95%) gave results of full-length homologs ~900 amino acids long (Fig. 4A; Table S8 at https://doi.org/10.6084/m9.figshare.22012931). All sequences share a homologous domain of 500 amino acids that aligns to position ~300 to 800 amino acids in protein VUZ27132.1.

10.1128/msystems.00119-23.5 FIG S4 Full uncollapsed clade 9 phylogenetic tree. Detailed version of Fig. 4C showing all sequences included in the analysis. Protein sequences from *Acetobacterium* spp. are highlighted in magenta. Circles at internal nodes indicate ultrafast bootstrap support values. Colored circles at the tip nodes indicate phylum. Download FIG S4, PDF file, 0.2 MB. Copyright © 2023 Kronen et al. 2023 Kronen et al. https://creativecommons.org/licenses/by/4.0/ This content is distributed under the terms of the Creative Commons Attribution 4.0 International license.

Phylogenetic analysis of protein VUZ27132.1 and its homologs revealed nine distinct clades (Fig. 4B). Some sequences are grouped into main lineages located within the clades by analysis of the genomic context and/or information from the literature about the protein’s enzymatic activity: (i) glutamate synthase β-subunit (GltD) homologs (35), (ii) *Archaea*-specific homologs, (iii) NADH-dependent reduced ferredoxin:NADP^+^ oxidoreductase large subunit (NfnB) homologs (36–39), (iv) dissimilatory sulfite reductase subunit L (DsrL) homologs (40–44), (v) NADPH-dependent oxidoreductase subunit C (NsoC) homologs (45) and 2-oxoglutarate:NADP^+^ oxidoreductase subunit α (KGOR-α) (46), (vi) the NfnA of Dehalogenimonas lykanthroporepellens, (vii) large homologs in *Proteobacteria*, (viii) a functionally and taxonomically heterogeneous group, and (ix) clade 9 homologs (Fig. 4B; Table S8 at https://doi.org/10.6084/m9.figshare.22012931). MAG ISORED-2 encodes another member of the ENOG4107QZ5 orthologous group (protein VUZ23049.1), but it clusters with the glutamate synthase β-subunit (GltD) sequences in clade 1 (Fig. 4A, II). The families *Desulfobacteraceae* and *Syntrophobacteraceae* contain an even longer ortholog (~1,300 to 1,400 amino acids) (Fig. 4A and B, clade 7).

The clade 9 homologs are defined as the most closely related to A. wieringae’s isoprene-regulated oxidoreductase (VUZ27132.1); with a few exceptions, they all aligned to the entire 901-amino-acid-sequence. They are mainly distributed among *Firmicutes* strains, but some are found in *Spirochaetes*, *Tenericutes*, and *Actinobacteria* as well as *Chloroflexi*, *Bacteroidetes*, and *Proteobacteria* (Fig. 4A; Table S8 at https://doi.org/10.6084/m9.figshare.22012931). Most taxa containing clade 9 homologs are strict anaerobes (see Table S8 at https://doi.org/10.6084/m9.figshare.22012931).

To determine uniqueness and conservation of protein VUZ27132.1 and the putative *isr* operon, a more detailed and extended phylogenetic tree of clade 9 sequences was generated, and a gene neighborhood network (GNN) analysis was performed. Other *Acetobacterium* strains also contain clade 9 proteins, which share ~47 to 49% amino acid sequence identity with protein VUZ27132.1 (Fig. 4C and Fig. S4; Table S9 at https://doi.org/10.6084/m9.figshare.22012931). Protein VUZ27132.1 from A. wieringae ISORED-2 is distinct from any other *Acetobacterium* homolog in a subclade (Fig. 4C, cluster I), while homologs from A. woodii, A. fimetarium, A. malicum (WP_186895649.1), A. paludosum, and A. tundrae are clustered together in a separate subclade (Fig. 4C, cluster II). Homologs from A. dehalogenans DSM 11527, *A*. sp. HGW-4, *A*. sp. UBA6819, A. malicum (WP 186893884.1), and *A*. sp. KB-1 are located in a third subclade (Fig. 4C, cluster III). Besides sharing proximity to *hypA* and *hypB*, the gene neighborhoods of clade 9 proteins in *Acetobacterium* spp. from clusters II and III are distinct from those of A. wieringae ISORED-2 (Fig. S5). In the case of cluster III (Fig. 4C), the oxidoreductase gene is flanked by another ferredoxin oxidoreductase (ENOG41061TH) and a helix-turn-helix domain-containing protein (ENOG4107MUM) (Fig. S5). Corresponding genes of the oxidoreductase homologs in cluster II are all flanked by a transcriptional regulator (TerR) and a hypothetical protein, but in the cases of A. woodii and A. fimetarium, an additional oleate hydratase is located between TerR and the hypothetical protein (Fig. 4C; Fig. S5).

10.1128/msystems.00119-23.6 FIG S5 Gene arrangements of isoprene-upregulated putative oxidoreductase IsrA from the A. wieringae ISORED-2 MAG and selected clade 9 homologs. Clade 9 proteins from two *Pelotomaculum* sp. are most closely related to IsrA. The gene environment of clade 9 homologs in other *Acetobacterium* spp. is different than A. wieringae ISORED-2. *Acetobacterium* spp. gene arrangements from cluster III (green underline; *A*. sp KB-1; A. malicum [WP 186893884.1]; A. dehalogenans Fig. 4C) are identical. Gene arrangements in *Acetobacterium* spp. from cluster II (blue and turquoise underline Fig. 4C) are very similar except that A. woodii and A. fimetarium also encode an oleate hydratase, and A. tundrae and A. paludosum do not. *Desulfobacteraceae* and *Syntrophobacteraceae* contain a longer homolog (~1,300 to 1,400 amino acids), which is not located next to *hypA* or *hypB*. The *Desulfobacteraceae* bacterium is shown as an example of how these gene arrangements are organized. Blank arrows indicate proteins of unknown function. Download FIG S5, PNG file, 0.4 MB. Copyright © 2023 Kronen et al. 2023 Kronen et al. https://creativecommons.org/licenses/by/4.0/ This content is distributed under the terms of the Creative Commons Attribution 4.0 International license.

Moreover, an alignment of all *Acetobacterium* clade 9 homologs (Fig. S6) revealed that protein VUZ27132.1 contains 26 unique amino acids at position 756 to 782, which cannot be found in any *Acetobacterium* homolog except A. wieringae strain Y and their two most similar homologs (Fig. 4C, cluster I). These two homologs (HBC92819.1 and WP_190259616.1) (Fig. 4C; Fig. S5 and S6) belong to *Pelotomaculum* spp. and share ~77% amino acid sequence identity with protein VUZ27132.1. However, the two *Pelotomaculum* sp. genomes do not encode the complete putative *isr* operon, missing the ferredoxin (ENOG4105DQ9) and the second *hypA* homolog (Fig. S5). GNN analysis results identified genome neighbors of 988 homologs from protein VUZ27132.1 (see Table S12 at https://doi.org/10.6084/m9.figshare.22012931): (i) hydrogenase/urease nickel incorporation, metallochaperone, hypA (“HypA,” PF01155, cooccurrence frequency of 0.73, average distance of 1.05); (ii) CobW/HypB/UreG, nucleotide-binding domain (“HypB,” PF02492, cooccurrence frequency of 0.56, average distance of 1.39); (iii) bacterial regulatory proteins, TetR family (“TetR_N,” PF00440, cooccurrence frequency of 0.21, average distance of 1.64); (iv) domain of unknown function (“DUF5692,” PF18948, cooccurrence frequency of 0.4, average distance of 2); and (v) none (69% of the proteins in UniProt are not associated with a Pfam family). Hence, no corresponding gene of the clade 9 proteins has a ferredoxin from the orthologous group ENOG4105DQ9 located adjacent to it apart from A. wieringae ISORED-2 itself and A. wieringae strain Y. Aside from these two *Acetobacterium* strains involved in isoprene reduction, the gene arrangement of the putative *isr* operon is not found in any other organism on NCBI.

10.1128/msystems.00119-23.7 FIG S6 Amino acid alignment of IsrA (VUZ27132.1), its closest homologs in *Pelotomaculum* sp., and clade 9 homologs in other *Acetobacterium* spp. Only amino acid positions 735 to 796 are shown. IsrA contains 26 unique amino acids that cannot be found in other *Acetobacterium* spp. and only in two clade 9 homologs most closely related to protein VUZ27132.1, which belong to *Pelotomaculum* sp. (Fig. 4C). The sequences were aligned with MAFFT-L-INS-i v7.407. The colors indicate amino acids of different biochemical properties as displayed in UGENE. Download FIG S6, PNG file, 0.4 MB. Copyright © 2023 Kronen et al. 2023 Kronen et al. https://creativecommons.org/licenses/by/4.0/ This content is distributed under the terms of the Creative Commons Attribution 4.0 International license.

### Predicted functional annotation of proteins encoded in the putative *isr* operon.

Functional annotation to predict domains and important sites of the proteins encoded by the putative *isr* operon was performed with InterProScan (47) (see Table S9 at https://doi.org/10.6084/m9.figshare.22012931).

Proteins VUZ27133.1 and VUZ27136.1 were predicted to consist entirely of a HypA domain (PF01155, IPR000688) Ni-metallochaperone. Like HypA proteins in other organisms, both HypA proteins in A. wieringae ISORED-2 contain conserved binding properties to Ni^2+^ (backbone amides of residues His2 and Glu3) and Zn^2+^ (two CxxC motifs) (Fig. S7) (48). HypA1 (VUZ27133.1) and HypA2 (VUZ27136.1) from A. wieringae ISORED-2 only share 25% amino acid sequence identity with each other. HypA1 and HypA2 share 25% and 24% amino acid sequence identity with well-characterized HypA from Helicobacter pylori, respectively (49).

10.1128/msystems.00119-23.8 FIG S7 Sequence alignment of HypA proteins. Like HypA proteins in other organisms, both HypA proteins in A. wieringae ISORED-2 contain conserved binding properties to Ni^2+^ (backbone amides of residues His2 and Glu3, highlighted in red) and Zn^2+^ (two CxxC motifs, highlighted in green). UniProtKB accession numbers are Helicobacter pylori (*HELPY*) HypA, P0A0U4; Escherichia coli (*ECOLI*) HypA, P0A700; and Bradyrhizobium japonicum (*BRAJP*) HypA, A0A1Y2JZ19. GenBank accession numbers are *A*. *wieringae* ISORED-2 HypA1 (VUZ27133.1) and HypA2 (VUZ27136.1) *A*. *wieringae* ISORED-2. Refer to reference 48 for details. Note that HypA1 and HypA2 from A. wieringae ISORED-2 only share 25% amino acid sequence identity with each other. HypA1 shares 25% and HypA2 shares 24% amino acid sequence identity with HypA from Helicobacter pylori. Alignment was performed with MAFFT-L-INS-i v7.407. Download FIG S7, PNG file, 0.1 MB. Copyright © 2023 Kronen et al. 2023 Kronen et al. https://creativecommons.org/licenses/by/4.0/ This content is distributed under the terms of the Creative Commons Attribution 4.0 International license.

Protein VUZ27134.1 belongs to the TIGR00073 family hydrogenase maturation factor HypB and is predicted to contain a CobW/HypB/UreG nucleotide-binding domain (PF02492). Within characterized HypB proteins, VUZ27134.1 is most similar to type 1 HypB proteins, which lack the N-terminal metal-binding region and only possess the G-domain cysteine Ni-binding residues (CHX_n_C motif; Cys106 and Cys142), like HypB from Helicobacter pylori (50, 51). However, H. pylori HypB proteins have an N-terminal extension (17 amino acids), which is missing for the HypB protein VUZ27134.1 (Fig. S8). HypB from A. wieringae ISORED-2 shares 42% amino acid sequence identity with well-characterized HypB from H. pylori.

10.1128/msystems.00119-23.9 FIG S8 Sequence alignment of HypB proteins. Like HypB proteins from other organisms, HypB from A. wieringae ISORED-2 contains the CobW/HypB/UreG nucleotide-binding domain (PF02492, highlighted in blue) and CHX_n_C motif involved in Ni^2+^ binding (Cys106 and Cys142; highlighted in red plus star). UniProtKB accession numbers are sequences of Helicobacter pylori (*HELPY*) HypB, O25560; Archaeoglobus fulgidus (*ARCFU*) HypB, O28903; Methanocaldococcus jannaschii (*METJA*) HypB, Q57884; Escherichia coli (*ECOLI*) HypB, P0AAN3; and Bradyrhizobium diazoefficiens (*BRADU*) HypB, Q45257. HypB can be classified into three types according to the sequence variations in the N-terminal extension. Type 1 HypB (e.g., HypB *HELPY*) contains a short N-terminal extension that does not bind metal (highlighted in purple). Type 2 HypB (e.g., HypB *ECOLI*) contains a CxxCGC motif (highlighted in orange) at the N terminus and a proline-containing linker region (highlighted in yellow). In type 3 HypB (e.g., HypB *BRADU*), the linker region is rich in histidine residues capable of binding multiple metal ions (highlighted in green). See reference 50 for details. HypB from A. wieringae ISORED-2 shares 42% amino acid sequence identity with HypB Helicobacter pylori. Alignment was performed with MAFFT-L-INS-i v7.407. Download FIG S8, PNG file, 0.3 MB. Copyright © 2023 Kronen et al. 2023 Kronen et al. https://creativecommons.org/licenses/by/4.0/ This content is distributed under the terms of the Creative Commons Attribution 4.0 International license.

The first ~270 amino acids of protein VUZ27132.1 and protein VUZ27135.1 align to each other (BLASTp ID of <26%), but no functional protein domain could be predicted from 1 to 270 amino acids by InterProScan or NCBI’s Conserved Domain Database. The remaining part of VUZ27135.1 holds a predicted 4Fe-4S ferredoxin-type, iron-sulfur-binding domain (IPR017896) (see Table S10 at https://doi.org/10.6084/m9.figshare.22012931), indicating the binding of a single [4Fe-4S] cluster.

**FIG 5:**
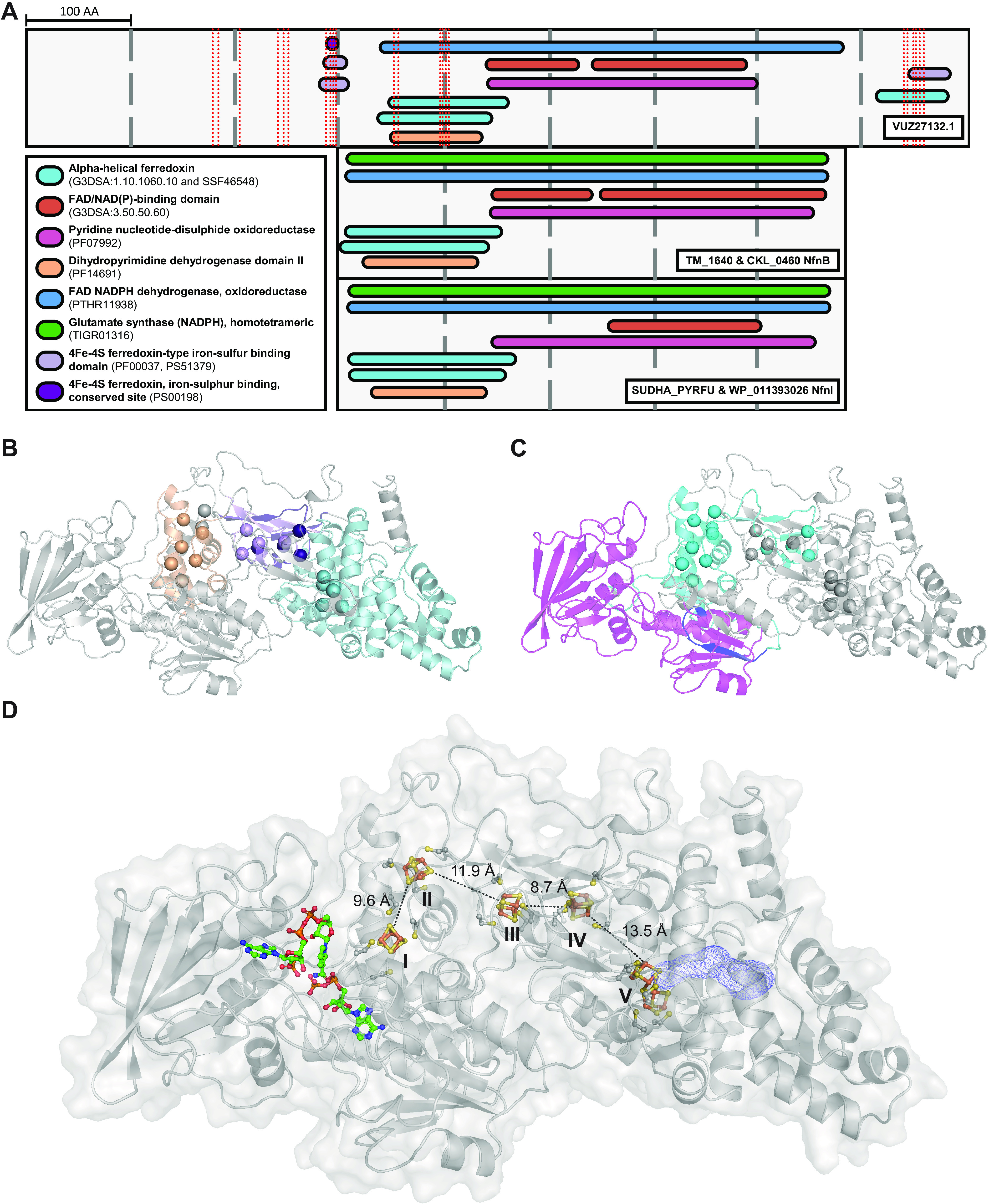
Protein domains of IsrA. (A) Domain comparison of IsrA and the characterized large subunits of Nfn (cysteine residues involved in binding iron-sulfur clusters are shown as red dashed lines). (B and C) Overlay of the IsrA-predicted domains onto its protein model. Cysteine sulfur atoms involved in binding the iron-sulfur clusters are shown as spheres. (D) Overview of IsrA with all cofactors. The dotted black line indicates the shortest distance between Fe/S clusters. Blue mesh on the right indicates the predicted tunnel for isoprene. Results of the InterProScan analysis are shown for each protein (detailed information is available in Table S9 at https://doi.org/10.6084/m9.figshare.22012931).

Protein VUZ27132.1 is predicted to have oxidoreductase activity (Gene Ontology term GO:0016491). According to InterProScan results, protein VUZ27132.1 contains a flavin adenine dinucleotide (FAD)/NAD(P)-binding domain (IPR023753, PF07992) as well as a dihydropyrimidine dehydrogenase domain II (IPR028261, PF14691), which carries two [4Fe-4S] clusters (Fig. 5A to C). Moreover, InterProScan results suggest that protein VUZ27132.1 contains two additional [4Fe-4S] ferredoxin-type iron-sulfur-binding domains (PS51379) (Fig. 5; see also Table S10 at https://doi.org/10.6084/m9.figshare.22012931) located at positions 289 to 300 and 848 to 877 amino acids. Examination of the protein model for VUZ27132.1, generated with ColabFold, corroborates the binding environment of these four canonical [4Fe-4S] clusters as {[4Fe-4S](Cys_4_)}, with the cysteine residues in near tetrahedral conformation, and all four [4Fe-4S] clusters laid out in a chain (Fig. 5D). In addition, six cysteine residues were found topologically close (Fig. 5B to D), with four of them forming two distinct CxC motifs (Cys180-Cys182 and Cys254-Cys256). This is a less common Fe/S cluster-binding motif but is still found in some proteins, such as ferredoxin:thioredoxin reductase (52) or ISCA2 (53). Distances between the Fe/S clusters in electron transfer chains are usually ~8 to 15 Å to adjacent clusters (52, 54, 55), which is concordant with the distances measured between [4Fe-4S] clusters in IsrA (8.7 to 11.9 Å) (Fig. 5D), including between clusters IV and V (13.5 Å), which further supports the presence of an Fe/S cluster at the cluster V site as part of an electron transfer chain.

## DISCUSSION

This study investigated the genetic basis for bacterial isoprene reduction activity previously observed in an isoprene-reducing enrichment (23). The bacteria in this enrichment culture were now identified as a *Comamonas* sp. and A. wieringae, named MAG ISORED-1 and ISORED-2, respectively. A. wieringae ISORED-2 dominates the isoprene-reducing culture (~89% relative abundance metagenome sequencing and ~94% relative biomass) (Table 1) and like other *Acetobacterium* spp. encodes the Wood-Ljungdahl pathway (WLP) for autotrophic growth (56), the Na^+^-translocating ferredoxin:NAD^+^ oxidoreductase (Rnf complex) (57), F_1_F_0_-ATPase (58), the electron transfer flavoproteins (59), and an electron-bifurcating [FeFe]-hydrogenase (60). Although A. wieringae ISORED-2 dominates the isoprene-reducing culture, a *Comamonas* sp., which shows highest sequence similarity to Comamonas aquatica CJG (78.9% ANI and 74.5% AAI) (61), is also present (~11% relative abundance metagenome sequencing and ~6% relative abundance biomass) (Table 1). However, its relative abundance in H_2_/HCO_3_^−^/isoprene-fed cultures (11%) was lower than in H_2_/HCO_3_^−^-fed cultures (~23%) based on coverage values from metagenome sequencing (Table 1), suggesting that these cells do not benefit from the inclusion of isoprene. Additionally, one of two proteins that were significantly more abundant following exposure to isoprene in *Comamonas* sp. ISORED-1 is SpoT (VUZ25726.1), a ppGpp synthetase/hydrolase, indicating that the cells are experiencing nutrient stress. Bacteria respond to nutritional stress by producing (p)ppGpp, which triggers a stringent response, resulting in growth arrest and reallocation of cellular resources (32, 62). In Escherichia coli, fatty acid starvation was found to induce (p)ppGpp accumulation synthesized exclusively by SpoT (63). SpoT interacts with acyl carrier protein (ACP) to likely induce a conformational switch that favors (p)ppGpp synthesis following fatty acid starvation (64). Interestingly, ACP was one of the significantly less abundant proteins in *Comamonas* sp. ISORED-1 in the presence of isoprene (Table 1), and proteins observed in the metaproteome included those for beta-oxidation of fatty acids (see Table S11 at https://doi.org/10.6084/m9.figshare.22012931). These results put forward that *Comamonas* sp. ISORED-1 is growing on necromass (e.g., fatty acids) and is experiencing stress in the presence of isoprene, which slows down cellular growth and metabolism via the (p)ppGpp stringent response. Taken together with the recent isolation of another isoprene-reducing A. wieringae strain (strain Y) (24) that shares high sequence similarities with A. wieringae ISORED-2 (99.5% ANI and 99.7% AAI) and is a pure isolate, it can be concluded that A. wieringae ISORED-2 is solely responsible for the isoprene reduction ability in this mixed enrichment culture.

Isoprene reduction was found to be an induced rather than constitutive trait, and comparative proteomics identified 13 significantly more abundant proteins following isoprene exposure. Apart from A. wieringae ISORED-2’s oxidoreductase (VUZ27132.1), no isoprene-responsive protein from A. wieringae ISORED-2 or *Comamonas* sp. ISORED-1 is predicted by protein function to be involved in redox processes (Table 2). This makes the oxidoreductase from the A. wieringae lineage the only likely candidate within the 13 isoprene-responsive proteins that could catalyze the isoprene hydrogenation reaction. The oxidoreductase is encoded in a putative five-gene operon together with the corresponding genes for three nickel-binding chaperones and one 4Fe-4S ferredoxin (Fig. 2A). Four out of the five proteins encoded in this putative operon were also significantly higher in abundance following isoprene exposure in A. wieringae ISORED-2 (Fig. 1) and are also found to be unique to the A. wieringae ISORED-2 genome by pangenomic comparison of selected available *Acetobacterium* genomes (see Table S7 at https://doi.org/10.6084/m9.figshare.22012931). Because the closest relative A. wieringae DSM 1911 did not exhibit isoprene-reducing activity (23), it follows that genes encoding isoprene reduction most likely sit within this unique set. Apart from the corresponding genes for 4Fe-4S ferredoxin, two HypA proteins, and the oxidoreductase (VUZ27132.1), no other genes responding to isoprene are unique to the ISORED-2 MAG. Henceforth the operon will be referred to putatively as the isoprene-regulated operon (*isr* operon) and the oxidoreductase (VUZ27132.1) as the putative isoprene reductase or IsrA (gene name *isrA*).

Potential “isoprene reductase” candidates have also been shortlisted in recently discovered isoprene-reducing A. wieringae strain Y (24). A total of 44 putative ene-reductases (ERs) in strain Y were designated by Jin *et al.* as IsoR (standing for isoprene reductase) based on enzyme functionality predictions, but these suggestions were not substantiated by their experimental data. Using proteomic analysis, Jin *et al.* identified a candidate ER (LNN31_08025, which shares 100% nucleotide sequence identity with IsrA) for the isoprene reduction reaction, but it is not specifically referred to as “the” isoprene reductase since all 44 putative ERs in strain Y are named “IsoR.” Like IsrA in A. wieringae ISORED-2, LNN31_08025 in strain Y is also encoded in a five-gene operon that has 100% nucleotide sequence identity with the *isr* operon in A. wieringae ISORED-2. Surprisingly, Jin *et al.* do not mention the operon nor the other proteins of the *isr* operon even though their proteomic data show significant abundance of all 4 proteins with LFC values among the highest in their data set (data set 3 in reference 24). Taken together, results from Jin *et al.* further validate that IsrA in A. wieringae ISORED-2 is the enzyme responsible for the isoprene hydrogenation reaction.

Based on domain predictions, IsrA contains a nested FAD and NAD(P)H binding site as well as two pairs of canonical [4Fe-4S] clusters (clusters I to IV, Fig. 5D) and one extra hypothesized Fe/S cluster in a Cys_6_-bonding environment (cluster V, Fig. 5D). The best-characterized and only crystallized proteins in the orthologous group of IsrA are the β-subunits of NADH-dependent ferredoxin-NADP^+^-oxidoreductases (Nfn) (Fig. 4A and B, evolutionary group 3). Nfn is an electron-bifurcating enzyme (65) composed of two subunits, NfnA (32.6 kDa) and NfnB (49.8 kDa). Crystal structures from Thermotoga maritima (TM_1640) and Pyrococcus furiosus (PF1327) revealed that NfnB contains two [4Fe-4S] clusters as well as binding sites for NADPH and FAD, with FAD being the site of electron bifurcation (38, 66, 67) (Fig. 5A). Since IsrA is predicted to contain a FAD/NAD(P)H binding site as well as five Fe/S clusters (four [4Fe-4S] clusters plus a putative fifth Fe/S cluster), and a 4Fe-4S ferredoxin (VUZ27135.1) is encoded in the putative *isr* operon, bifurcation may be a reaction mechanism to contemplate for IsrA. The standard redox potential of the isoprene/methylbutene couple is not known but based on calculation using the estimation of isoprene energy of formation 197 kJ mol^−1^ (23, 68, 69) and theoretical stoichiometries with H_2_ as an electron donor for the isoprene hydrogenation reaction (68, 70),

(1) $$H_{2}\;+\;C_{5}H_{8}\to C_{5}{\text{H}}_{\text{1}0}\;\;\;\;\;\;\;\Delta G^{0}=-\text{137 kJ}/{\text{mol}}^{-\text{1}},$$

the standard reduction potential (E^0^) of the isoprene/methylbutene couple calculated using the Nernst equation is estimated as

$$E^{0}=+\Delta G^{0}/\text{nF}$$

(2) $$E^{0}=\left(-\text{137,}000\text{ J}/\text{mol}\right)÷\left(-\text{2 mol }\times \text{ 96,484 J}/\text{mol V}\right)=\text{ 7}0\text{9 mV}$$

The Nernst equation for standard potential of biological systems at pH 7 is

$$\text{Eox/red }=E^{0}\text{ox/red }+\left(2.3\text{ RT/nF}\right)\text{ log }\left[\text{ox}\right]/\left[\text{red}\right],$$

where 2.3 RT/F = 0.059 at *T* = 289 K, *F* = 96,500, and *R* = 8.31, yielding

$$\text{Eox/red}=E^{0}\text{ox/red }+\left(0.059\text{/n}\right)\text{ log }\left[\text{ox}\right]/\left[\text{red}\right].$$

The standard electron potential of the hydrogen electrode is EH_2_ = E^0^H_2_ + (0.059/1) log (H^+^); E^0^H_2_ = 0; EH_2_ = −0.059 pH

(3) $$E^{0\prime }=E^{0}\;-\;0.0\text{59 pH}=0.709\;V\;-\;\left(0.059\times 7\right)=+\text{ 296 mV}.$$

Hypothetically, similar to caffeate reduction, NADH (E^0′^ = −320 mV) derived from the [FeFe]-hydrogenase and Rnf complex could act as the reductant for the exergonic reduction of isoprene to methylbutene (E^0′^ = +296 mV), which is coupled to the endergonic reduction of ferredoxin (E^0′^ = −420 mV) (71). As with caffeate respiration, the reduced ferredoxin could be reoxidized at the Rnf complex to generate an Na^+^ gradient (72). Out of 12 known flavin-based bifurcating enzymes (65, 71), three are found in *Acetobacterium* spp.: the bifurcating [FeFe]-hydrogenase (60), lactate dehydrogenase/electron transfer flavoprotein (Bf-Ldh) (73), and the caffeyl-coenzyme A (caffeyl-CoA) reductase (72). However, homology to bifurcating enzymes is not sufficient to guarantee electron bifurcating functionality (65), but as energetics and the binding sites of IsrA support the idea of an electron bifurcating process, it should be considered in future biochemical investigations of IsrA. If isoprene reduction was a linear process, reduction would have to be coupled to ATP synthesis through establishment of an ion gradient since the reduction of isoprene conserves energy (23). In contrast, Jin *et al.* found no difference in acetate amounts between A. wieringae strain Y cultures with and without isoprene, concluding that strain Y cannot conserve energy from isoprene reduction but cometabolizes isoprene. Jin *et al.* measured acetate amounts during transformation of only 100 μmol of isoprene (4 days), whereas in our previous study, acetate was measured during transformation of 800 μmol of isoprene (30 days). The difference in acetate amounts was only seen after the transformation of at least 500 to 600 μmol of isoprene (see Fig. 7 in reference 23). Until comparable results of this experiment are published, isoprene reduction is suggested to be coupled to energy conservation.

Beside IsrA, the putative *isr* operon contains three *hyp* genes (two *hypA* and one *hypB*; hydrogenase pleiotropic), which encode metallochaperones typically responsible for acquisition and insertion of nickel during maturation of [NiFe]-hydrogenases (74–78). The role of HypA and HypB during [NiFe]-hydrogenase maturation is well studied and first involves the metal-dependent (Ni^2+^) dimerization of HypB with one equivalent of nickel per dimer (79, 80). Second, the hydrolysis of GTP facilitates the transfer of Ni^2+^ from HypB to HypA by weakening the binding affinity of Ni^2+^ to HypB and promoting the formation of the HypAB heterodimer (81) or, in some organisms such as Thermococcus kodakarensis, a heterotetramer HypAABB (82). After the Ni^2+^ is transferred to HypA, HypA dissociates from the complex and delivers the cofactor to the large subunit of the hydrogenase (50, 83). Biosynthesis and maturation of the [NiFe]-hydrogenase active site is a complex multistep process also involving a number of other accessory Hyp proteins (HypCDEF) (84, 85). Yet, the genome of A. wieringae ISORED-2 does not harbor *hypCDEF* nor the core structural genes of the [NiFe]-hydrogenase (i.e., large and small [NiFe]-hydrogenase subunits), questioning the presence of three Hyp proteins in the *isr* operon. The Hyp proteins could potentially be involved with other Ni-dependent enzymes, since the genome of A. wieringae ISORED-2 encodes three other known Ni-containing enzymes: two lactate racemases (86), ISORED2_01724 (VUZ23303.1) and ISORED2_03140 (VUZ26077.1) and the CO dehydrogenase/acetyl-CoA synthase complex (CODH/ACS) (87) at ISORED2_03659 to ISORED2_03664, with an additional beta subunit encoded by ISORED2_01878. However, these enzymes are located in operons that include their own metallochaperones/maturation proteins, that is, *larE* (ISORED2_00963 and ISORED2_01065) (86) and *acsF* (ISORED2_03663) (87), respectively. This suggests that the two HypA and/or HypB proteins from A. wieringae ISORED-2 are unlikely to be involved with other Ni-containing enzymes but rather facilitate nickel insertion into an active site of one of the proteins in the *isr* operon. Genome neighborhood computation results show that out of 988 IsrA homologs, 73% are encoded within 5 genes to HypA and 56% are to HypB whereas no association of HypA or HypB could be found with the 4Fe-4S ferredoxin (see Table S12 at https://doi.org/10.6084/m9.figshare.22012931). This suggests that IsrA is the target protein for potential nickel acquisition and that this type of HypA and HypB protein may be required for the enzymatic function of many of these clade 9 oxidoreductase homologs. Known nickel-binding sites in Ni-containing proteins involve cysteine, histidine, or acidic residues, and in catalytic nickel-containing proteins, the nickel is in the site closest to the substrate (88). Prediction for substrate transport channels into IsrA suggests a tunnel that would terminate in the immediacy of the hypothesized site of Fe/S cluster V (Fig. 5D), which would hence be closest to isoprene. Thus, Fe/S cluster V could act as a potential nickel-binding site in IsrA since in other Ni-containing enzymes, Ni^2+^ is found closest to the substrate (e.g., [NiFe]-CODH/ACS and [NiFe]-hydrogenase) (88). In the [NiFe]-CODH and ACS, nickel is either next to the Fe/S cluster or substituting an Fe atom in the cluster, while in the [NiFe]-hydrogenase, it located close to the Fe of the Fe(CN)_2_CO group (88). Based on the protein model, the binding site of cluster V consists of six cysteine residues and has certain conformational resemblances with the Fe/S cluster binding sites in two other enzymes; on one hand, it resembles cluster A of the acetyl-CoA synthase (a Ni-Ni-[4Fe-4S] cluster) (89–91), and, on the other hand, it resembles the site of the P-type cluster in nitrogenases (55) or the similar double cubane [8Fe-9S] clusters (92). Interestingly, enzymes containing double cubane clusters have been shown to reduce small molecules such as acetylene to ethylene (92). Whether Fe/S cluster V does indeed accommodate the nickel-binding site in IsrA or not requires further biochemical characterization.

Promotor region analysis of the *isr* operon also supports the idea that IsrA might be a metal-dependent enzyme; four transcription factor (TF)-binding sites could be identified 13 bp upstream of the *isrA* gene start codon (Fig. S2). These are suggestive of binding sites for a ferric uptake regulator (Fur) or Ni(II)-dependent transcriptional regulator (NikR) type of TF (93, 94). As NikR is not encoded in A. wieringae ISORED-2, it is more likely that Fur regulates the operon (ISORED2_03031). A. wieringae ISORED-2 encodes multiple nickel import systems. NikA (nickel transport system permease) (95) and related subunits are mainly on contig ISORED2_43 (ISORED2_01669, ISORED2_03327, ISORED2_03336, ISORED2_03347, and ISORED2_03353). The lactate racemases have specific nickel importers as part of their operon(s): a three-component ATP-binding cassette (ABC) transporter *lar*(*MN*)*QO* (86) (ISORED2_00944-00946). Export of heavy metals from cells can be performed by a diverse number of mechanisms (96). While no Ni-specific metal exporters (97) were detected in the genome of A. wieringae ISORED-2, three P-type IB ATPases were found: ISORED2_00744 (*cadA*: Cd^2+^, Zn^2+^, and Co^2+^), ISORED2_02650 (*copA*: Cu^+^), and ISORED2_03115 (*ziaA*: Zn^2+^). Members of the P-type ATPase subfamily IB normally transport soft Lewis acids but often have limited specificity. Based on this, CadA or ZiaA might be the best candidates for P-type ATPase Ni^2+^ export given that CopA transports monovalent copper. The nickel import/export systems responsible for maintaining nickel homeostasis in A. wieringae ISORED-2 are yet to be identified, but since the main enzyme complex in the WLP (56, 98), the CO dehydrogenase/acetyl-CoA synthase, is a nickel-dependent enzyme (99, 100) and part of *Acetobacterium*’s core metabolism, it is expected that nickel homeostasis would be well maintained in A. wieringae ISORED-2.

Homologs of IsrA are widely distributed among anaerobic bacteria, but the putative *isr* operon, as observed in A. wieringae ISORED-2 and in A. wieringae strain Y, was not found in any other genome in NCBI. Acquisition of the putative *isr* operon via horizontal gene transfer may be one possible scenario that explains why only these two strains harbor the putative *isr* operon. The operon is located in a 44-kbp genomic region containing metabolic genes and is also flanked by mobile genetic elements (Fig. 3), a *Siphoviridae* provirus, and a series of insertion sequences in tandem, which suggests that the putative *isr* operon is placed in a dynamic genomic region of Acetobacterium wieringae ISORED-2. Other organisms that also encode the complete putative *isr* operon, from where horizontal gene transfer could have occurred, are yet to be identified.

Homologs of IsrA observed in other *Acetobacterium* spp. share only ~47 to 49% amino acid sequence identity, and these homologs are located in separate subclades (Fig. 4C) and their corresponding genes are found in different gene arrangements (Fig. S5). Together with the inability of other *Acetobacterium* spp. (i.e., A. woodii DSM 1030, A. malicum DSM 4132, A. wieringae DSM 1911, and A. dehalogenans DSM 11527) to reduce isoprene, as determined experimentally, the phylogenetic analysis provides further evidence that IsrA and its homologs in other *Acetobacterium* spp. have distinct enzymatic functions. Potential enzymatic functions to consider for IsrA homologs are the hydrogenation of unfunctionalized (conjugated) C = C bonds in other unsaturated hydrocarbons that are present in anoxic environments. For example, Jin *et al.* found that strain Y could, besides isoprene, also reduce 1,3-butadiene to 1-butene (24). 1,3-Butadiene is an anthropogenic compound used mainly to produce polymers (101) entering the environment via combustion processes and industrial releases (102, 103). Naturally occurring substrates to consider for the IsrA homologs could be terpenes (e.g., monoterpenes [C_10_H_16_] α-pinene, β-pinene, limonene, *trans*-β-ocimene, α-terpinene, myrcene, and sabinene), which consist of isoprene building blocks. This might be the case for Pelotomaculum schinkii, which encodes the most closely related homolog to IsrA (Fig. 4C). *P. schinkii* is a strictly anaerobic, syntrophic bacterium known to live in electron acceptor-depleted environments and metabolizes propionate and must resort to using H^+^ and CO_2_ as electron sinks (104). Degradation of propionate is thermodynamically challenging and can only be reached if H_2_ or formate are kept at very low concentrations by a syntrophic partner methanogen (104). However, due to its IsrA homolog, *P. schinkii* might have the ability to use unfunctionalized (conjugated) C = C bonds in unsaturated hydrocarbons as electron acceptors, which could enable them to grow axenically. As a general example, the oxidation of propionate coupled to isoprene reduction would be thermodynamically favorable (23, 68):

$$C_{3}H_{6}O_{2}\;+{\text{ 3 H}}_{2}O\;+{\text{ 3 C}}_{5}H_{8}\to {\text{HCO}}_{3}{}^{-}\;+{\text{ CH}}_{3}\text{COOH }+\;H^{+}+{\text{ 3 C}}_{5}H_{10}\;\;\;\;\Delta G^{0}=-{\text{316 kJ mol}}^{-\text{1}}.$$

This study provides evidence for the existence of a putative isoprene reductase. The putative isoprene reductase is of particular interest because of its reduction of an unfunctionalized conjugated C = C bond. IsrA homologs are widespread among various taxonomic groups of strictly and facultatively anaerobic bacteria (*Firmicutes*, *Spirochaetes*, *Tenericutes*, *Actinobacteria*, *Chloroflexi*, *Bacteroidetes*, and *Proteobacteria*), suggesting that the use of unfunctionalized C = C bonds in unsaturated hydrocarbons as anaerobic electron acceptors is a form of bacterial energy harvesting not previously recognized. While more rigorous physiological/biochemical testing is required to fully understand what the functions of IsrA and its homologs are, the results have environmental relevance in the context of furthering our understanding of electron sinks in anaerobic environments and furthering our understanding of contributing mechanisms to global isoprene turnover.

## EXPERIMENTAL PROCEDURES

### Strains and culturing conditions.

*Acetobacterium* species A. woodii DSM 1030, A. malicum DSM 4132, A. wieringae DSM 1911, and A. dehalogenans DSM 11527 were obtained from Deutsche Sammlung von Mikroorganismen und Zellkulturen (DSMZ, Germany).

Isoprene-reducing biomass was grown on H_2_/HCO_3_^−^/±isoprene, as described previously (23). Isoprene and H_2_ were resupplied every 2 days (RT-PCR and proteomics and cell suspension assays). After 4 (RT-PCR) or 10 (proteomics and cell suspension assays) days of incubation at 30°C, cells were harvested. Isoprene and methylbutene were quantified by gas chromatography (GC) using a GasPro Plot column (60 m × 0.32 mm, Agilent Technologies), as previously described (23).

### Cell suspension assays.

Cells from six flasks of H_2_/HCO_3_^−^/isoprene-grown cultures and six flasks of H_2_/HCO_3_^−^-grown cultures were pooled in an anaerobic chamber by pipetting cell aggregates into two separate 6-mL anoxic glass flasks. Cells were washed in minimal medium containing 1 mM titanium citrate (23), and optical density at 600 nm (OD_600_; 7.5) and volumes (1.57 mL) were adjusted between the two samples. Flasks were crimp sealed and flushed with N_2_ for 30 min to remove isoprene, methylbutenes, and CO_2_. Headspace was measured for isoprene and methylbutene before the experiment was started. H_2_ (7 × 10^4^ Pa), HCO_3_^−^ (60 mM), and isoprene (1 mM) were added, and cells were incubated at 30°C (with shaking at 180 rpm). Headspace (100 μL) was analyzed for isoprene depletion and methylbutene production as previously described (23). Liquid samples (0.04 mL) were analyzed for acetate. Acetate was analyzed as its ethyl ester derivative by GC-flame ionization detector (GC-FID) as previously described (23) but with reduced sample size.

### DNA extraction and Illumina sequencing.

DNA was extracted from isoprene-reducing cultures anaerobically grown on H_2_/HCO_3_^−^/±isoprene as described previously (23). Libraries were prepared using a Nextera XT DNA sample preparation kit according to the manufacturer’s protocol (Illumina). Sequencing reactions were carried out using MiSeq v2 (2 × 150 bp) chemistry (Illumina) on a MiSeq instrument (Illumina) at the Ramaciotti Centre for Genomics at University of New South Wales (UNSW; Sydney, Australia).

### RNA extraction and reverse transcription-PCR.

Cell aggregates from three flasks were pooled, centrifuged at 10,000 × *g* for 10 min, and disrupted in lysis buffer (400 μL) (105) with mechanical agitation (30 Hz for 10 min) in FastPrep lysis matrix A tubes (MP Biomedicals). RNA was extracted with sequential phenol-chloroform-isoamyl alcohol (25:24:1; pH 4.5), 3 M sodium acetate (pH 5.2), and chloroform treatments, precipitated with isopropanol and GlycoBlue coprecipitant (Thermo Fisher Scientific, Australia), resuspended in 35 μL of water, and stored at −20°C. Residual DNA in RNA samples was digested with RNase-free DNase (Qiagen) I and cleaned three times on a spin column from a PureLink RNA minikit (Thermo Fisher Scientific, Australia). RNA was quantified with a Qubit RNA high-sensitivity assay kit (Thermo Fisher Scientific, Australia). RNA samples were stored at −80°C until use. First-strand cDNA was synthesized from 100 ng of DNase I-treated total RNA using random hexamer primers from the RevertAid first-strand cDNA synthesis kit (Thermo Fisher Scientific) following the manufacturer’s instruction. In a negative control, the M-MuLV reverse transcriptase was replaced with water. Synthesized cDNA was used as the template in PCR with the Q5 high-fidelity DNA polymerase (New England BioLabs) using intergenic region primers (see Tables S3 and S4 at https://doi.org/10.6084/m9.figshare.22012931). Chromosomal DNA was used as template for the positive control (Fig. 2C).

### Protein extraction and LC-MS/MS analysis.

Cells were grown in 8 flasks with H_2_/HCO_3_^−^/isoprene and 8 flasks with H_2_/HCO_3_^−^. To increase cell mass, cells from two flasks were pooled from 8 to 4 samples for each condition, that is, 4 replicates for each condition. Cell aggregates were transferred into 2-mL tubes inside the anaerobic chamber, centrifuged at 10,000 × *g* for 10 min, and stored at −20°C until use. Harvested cells suspended in 100 μL of lysis buffer (105) were mechanically disrupted in FastPrep lysis matrix A tubes (MP Biomedicals) at 30 Hz for 10 min. Crude extracts were passed through a 30-kDa Amicon Ultra 0.5-mL centrifugal filter and washed 6 times with 200 μL of 50 mM NH_4_HCO_3_ buffer (pH 6.9). Protein concentrations were determined with the Quick Start Bradford protein assay following the manufacturer’s instructions (Bio-Rad Laboratories, Australia) and adjusted to 2 μg μL^−1^; 10 μL (20 μg) was used for filter-aided sample preparation (FASP) (106–108). Samples were treated with 5 mM dithiotreitol (DTT) at 37°C for 30 min. Protein lysates were then transferred to 30-kDa Amicon Ultra 0.5-mL centrifugal filters and treated following the FASP method involving an alkylation step (100 μL of 50 mM iodoacetamide). Trypsin solution (1 μL of a 200 ng μL^−1^ stock) was added for digestions at 37°C overnight. Peptides were eluted in 2 × 20 μL 50 mM NH_4_HCO_3_ buffer and stored at −20°C until LC-MS/MS analysis.

Sample analysis was performed at the Bioanalytical Mass Spectroscopy Facility (BMSF) at UNSW. Digested peptides were separated by nanoLC using an Ultimate nanoRSLC ultraperformance liquid chromatography (UPLC) and autosampler system (Dionex, Amsterdam, Netherlands). Samples (2.5 μL) were concentrated and desalted onto a micro C_18_ precolumn (300 μm × 5 mm, Dionex) with water:acetonitrile (98:2, 0.1% trifluoroacetic acid [TFA]) at 15 μL/min. After a 4-min wash, the precolumn was switched (Valco 10-port UPLC valve, Valco, Houston, TX) into line with a fritless nano column (75 μm × ~15 cm) containing C_18_AQ medium (1.9 μm, 120 Å, Maisch, Ammerbuch-Entringen, Germany). Peptides were eluted using a linear gradient of H_2_O:CH_3_CN (98:2, 0.1% formic acid) to H_2_O:CH_3_CN (64:36, 0.1% formic acid) at 200 nL/min over 30 min. High voltage (2,000 V) was applied to low-volume titanium union (Valco), and the tip was positioned ~0.5 cm from the heated capillary (*T* = 275°C) of an Orbitrap Fusion Lumos (Thermo Electron, Bremen, Germany) mass spectrometer. Positive ions were generated by electrospray, and the Fusion Lumos was operated in data-dependent acquisition mode (DDA).

A survey scan *m*/*z* 350 to 1,750 was acquired in the orbitrap (resolution = 120,000 at *m*/*z* 200, with an accumulation target value of 400,000 ions) and lockmass enabled (*m*/*z* 445.12003). Data-dependent tandem MS analysis was performed using a top-speed approach (cycle time of 2 s). MS2 spectra were fragmented by high-energy collisional dissociation (HCD; Normalised Collision Energy [NCE] = 30) activation mode, and the ion trap was selected as the mass analyzer. The intensity threshold for fragmentation was set to 25,000. A dynamic exclusion of 20 s was applied with a mass tolerance of 10 ppm.

### Genome assembly and annotation.

Quality trimming was performed with BBDuk (http://sourceforge.net/projects/bbmap/). Filtered reads were coassembled with MegaHIT v1.1.3 (109) and default parameters. Contigs ≥2.5 kbp were manually binned and curated under anvi’o v5.2.0 (110). The contig containing the rRNA operon was removed due to its chimeric nature (a single chimeric contig was detected). MAG completion estimates were obtained with (i) anvi’o bacterial Single Copy Gene (SCG) profile, (ii) CheckM v1.1.2 (111) lineage_wf, and (iii) CheckM lineage_wf with domain-specific profiles. Protein-coding genes of the metagenome and genomes were predicted with Prodigal v2.6.3 (112).

Metagenome-assembled genomes (MAGs) derived from binning were identified and named based on the Genome Taxonomy Database with GTDB-Tk v0.1.3 (113). Predicted proteins were annotated with InterProScan v5.25-64 (114). Results were parsed with a custom script, iprs2anvio.sh (https://github.com/xvazquezc/stuff/blob/master/iprs2anvio.sh), and integrated in the anvi’o workflow. Predicted proteins were assigned to bacterial orthologous groups using the bactNOG database from EggNOG v4.5.1 (115) with EggNOG-mapper v1.0.3-3-g3e22728 (115). Genome annotation was performed with a modified version of Prokka v1.13.3 (116), in which Prodigal generates partial gene calls at the ends of contigs to minimize differences between Prokka- and anvi’o-based gene predictions.

Prophage/provirus prediction was performed with VirSorter v1.0.6 (117), PHASTER web server (118), Phigaro v2.3.0 (119), and CheckV v0.7.0 with the v0.6 database (120).

### Refinement of the operon gene environment.

Due to the high coverage of ISORED-2, only reads from the samples containing isoprene were mapped back to the ISORED-2 MAG with Bowtie2 v2.3.4.3 (121) and examined in Integrative Genomics Viewer (IGV) v2.8.10 (122). The genome assembly graph was visualized with Bandage v0.8.1 (123). ISORED-2 was iteratively reassembled with MIRA v5rc2 (https://github.com/bachev/mira).

### Pangenome analysis.

Pangenomic analysis of the genus *Acetobacterium* with eight reference *Acetobacterium* genomes (see Table S5 at https://doi.org/10.6084/m9.figshare.22012931) was performed with anvi’o v5.2.0 (110) following the standard pangenomics workflow (http://merenlab.org/2016/11/08/pangenomics-v2). Genes were clustered with MCL inflation values of 2, 4, and 6 (124). Gene clusters were grouped based on their presence in all 9 genomes (core), at least 7 out of 9 (soft core), at least 4 out of 9 genomes (shell), or unique to the organism (singleton). Average nucleotide identity (ANI) between *Acetobacterium* genomes was calculated with pyani v0.2.7 (125). Average amino acid identity was calculated with CompareM v0.0.23 (https://github.com/dparks1134/CompareM).

### MS data analysis.

The raw MS data were processed using MaxQuant software (version 1.6.2.1) (126) and searched against a custom database of all predicted proteins in the metagenome of the isoprene-reducing culture (6,517 sequences). Enzyme specificity was set to trypsin/P, cleaving C terminus to lysine and arginine, and a maximum number of two missed cleavages allowed. Carbamidomethylation of cysteine was set as a fixed modification, and oxidation of methionines and acetylation of protein N termini were set as variable modifications. The minimum peptide length was set to 7 amino acids, and a maximum peptide mass was 4,600 Da. The minimal score for modified peptides was 40, and the minimal delta score for modified peptides was 6. Peptide intensities were normalized using MaxLFQ (127). Downstream analysis was performed in R v3.5.1 with the package DEP v1.4.0 (128). First, MaxQuant output data were filtered, retaining only proteins detected by at least two unique peptides and detected in all replicates. To reduce the influence of the changing community composition and their relative contributions toward the total metaproteomic data, the metaproteomic data were partitioned based on the source MAG and analyzed separately in DEP as follows. Label-free quantification (LFQ) intensities were normalized with vsn (129), and missing values were imputed by left-censored imputation (MinProb function). Differential expression analysis was conducted with limma (130). Proteins were considered differentially expressed if they had an adjusted false-discovery rate (FDR) *P* value of ≤0.05 and a log_2_ fold change (LFC) of ≥2 or ≤−2.

The numbers of peptide spectrum matches per protein were used to quantify the biomass contribution of each organism to the community (131).

### Phylogenetic analyses.

**(i) Molybdopterin oxidoreductase VUZ27132.1 (putative isoprene reductase).** Phylogenetic trees were constructed based on all protein sequences from the EggNOG database v4.5.1 (115) matching the orthologous group of VUZ27132.1 (ENOG4107QZ5) and its archaeal homolog (arCOG01292). Additional ENOG4107QZ5 sequences from other *Acetobacterium* genomes and characterized enzymes from the literature were included. An additional 1,000 top hit sequences to VUZ27132.1 retrieved from NCBI (BLASTp search on 10 December 2018) were clustered with CD-HIT v4.6 (-s 0.8 -c 0.8) and added to the data set (132). All sequences were aligned using MAFFT v7.313 (mafft-linsi) (133). The alignment was manually trimmed to restrict the phylogeny to the core/conserved region of the proteins, equivalent to positions 158 to 779 of 901 residues in VUZ27132.1. In addition, gap-rich columns were removed from the manually trimmed alignment with BMGE v1.12 (-m BLOSUM30 -g 0.9 -h 1) (134). The phylogenetic protein tree was constructed with IQ-TREE v1.6.7 (135) using a LG+I+G4 model (136) and 10,000 ultrafast bootstrap replicates (137). Trees were visualized using iTol interactive tree of life https://itol.embl.de/tree/4918010318257311557452600 (https://itol.embl.de/).

**(ii) Clade 9 phylogeny.** Additional top 1,000 records from NCBI were retrieved on 16 April 2021 to recover recently deposited IsrA (VUZ27132.1) orthologs. Only sequences with ≥50% identity and ≥75% coverage were added to the original data set, and identical sequences were removed. Orthologs from recently sequenced *Acetobacterium* genomes were also included (138). A total of 1,063 sequences were aligned with MAFFT-L-INS-i v7.407 (139). The resulting alignment was trimmed in BMGE v1.2 with permissive options (-m BLOSUM30 -g 0.9 -h 0.9) (134). The tree was inferred with IQ-TREE v2.1.2 (140) under the EX_EHO+R10 substitution model (141) and 1,000 ultrafast bootstrap replicates with nearest neighbor interchange optimization (––bnni) (142).

Gene neighborhoods of clade 9 proteins were examined with EFI-GNT (143) (search performed on 18 June 2021).

### Protein modeling.

The protein model of IsrA was generated with AlphaFold2 (v2.2.1) (144) as implemented in ColabFold v1.3.0-a2b37c (145). Modeling was run with the ––recompile-all-models option and AMBER relaxation.

Ligand binding sites for FAD and NADPH were predicted with the COACH-D webserver (146). Prediction of ligand channels was performed on the Caver Web v1.2 (147).

### Generation of genome neighborhood network.

Genome neighborhood networks (GNNs) for proteins from the putative *isr* operon (VUZ27132.1 to VUZ27136.1) were generated using the tools available on EFI—Enzyme Function Initiative Website (https://efi.igb.illinois.edu/efi-gnt/) (143, 148–150).

First, each of the protein sequences was subjected to a BLAST search against UniProt to search for homologous proteins using EFI-EST with the following parameters: E value of 1 × 10^−5^, maximum number of sequences retrieved of 1,000, and “Superkingdom: Bacteria,” “Superkingdom: Archaea,” and “Superkingdom: Eukaryota” as taxonomy filters. The corresponding sequence similarity network (SSN) was then generated with an alignment score threshold of 30%. The SSN was used as input to generate a GNN with a neighborhood size of 5 and a cooccurrence lower limit of 20%. The SSN cluster hub nodes from the GNN outputs are listed in Table S12 (at https://doi.org/10.6084/m9.figshare.22012931) with their cooccurrence values. From the cooccurrence values, users can identify how often neighbor genes occur next to the query (148).

### Data availability.

Raw sequencing data and annotated MAGs have been deposited in ENA under project PRJEB30289 (ERP112722). Metaproteomic data are available at the PRIDE database (PXD023683). Supplementary tables are available at FigShare (https://doi.org/10.6084/m9.figshare.22012931).
