# Supplementary figures and images for: Evidence for a Putative Isoprene Reductase in Acetobacterium wieringae
Source: mSystems. 2023 Mar 21;8(2):e00119-23. doi: 10.1128/msystems.00119-23 (PMC10134865; doi:10.1128/msystems.00119-23)

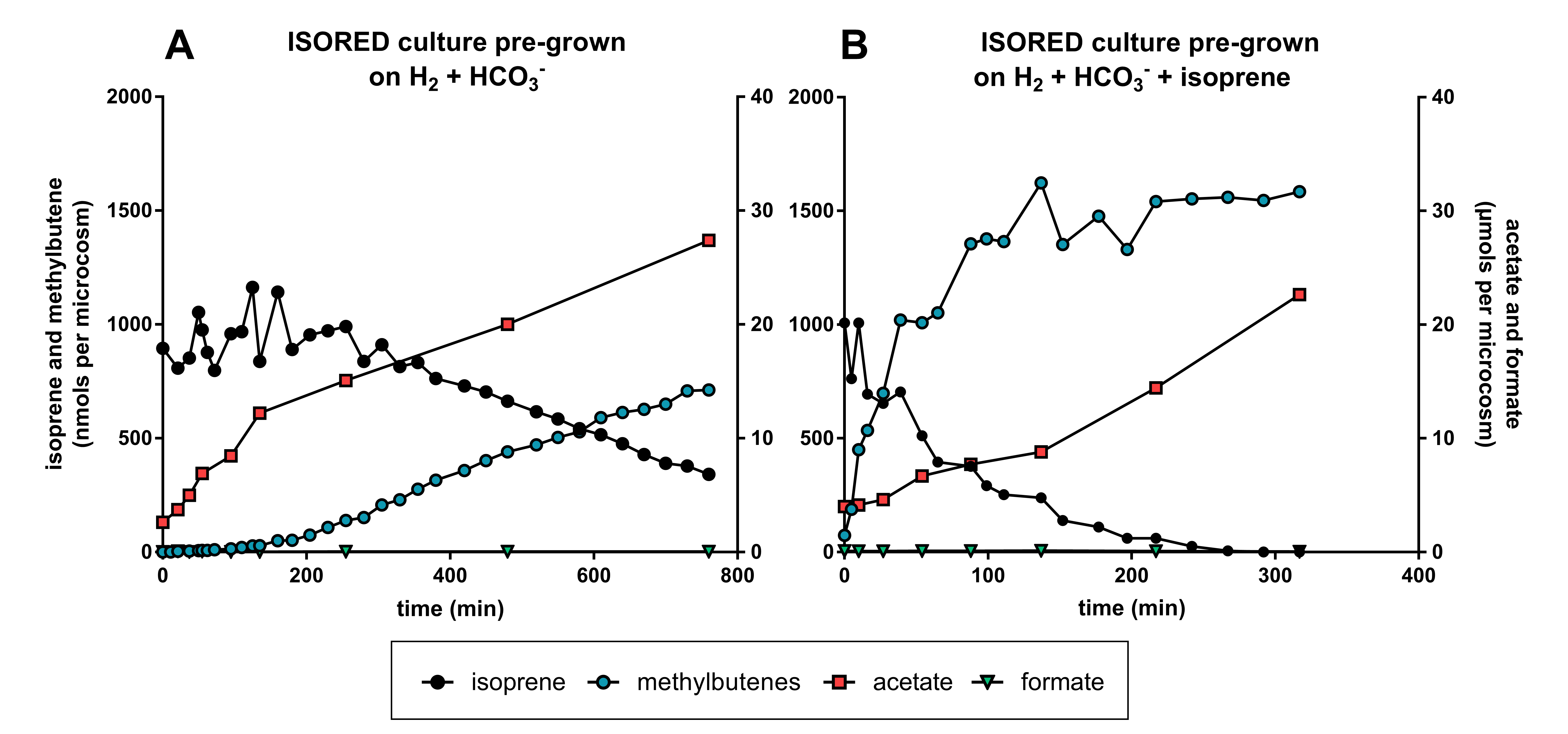

Supplement: FIG S1 [file msystems.00119-23-s0002.png]

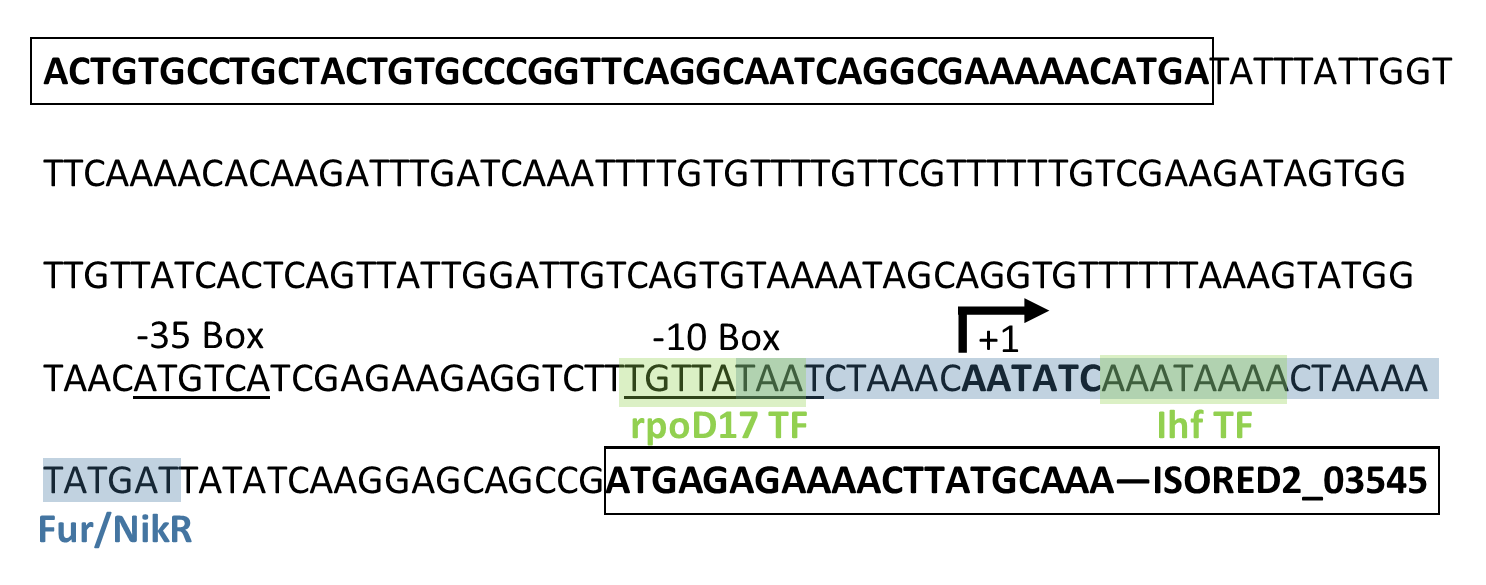

Supplement: FIG S2 [file msystems.00119-23-s0003.png]

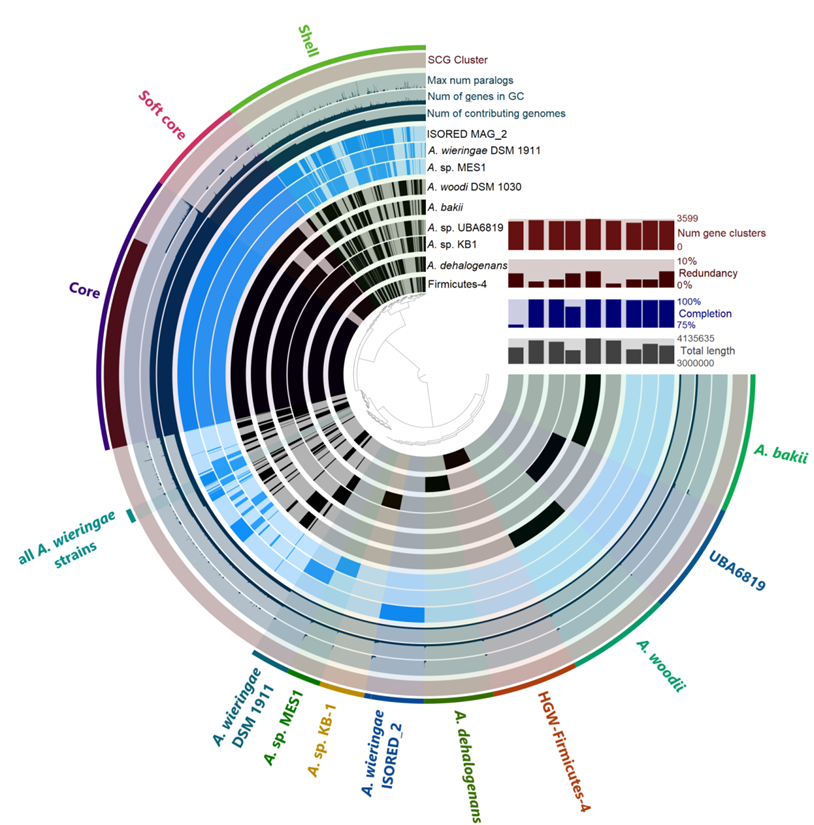

Supplement: FIG S3 [file msystems.00119-23-s0004.png]

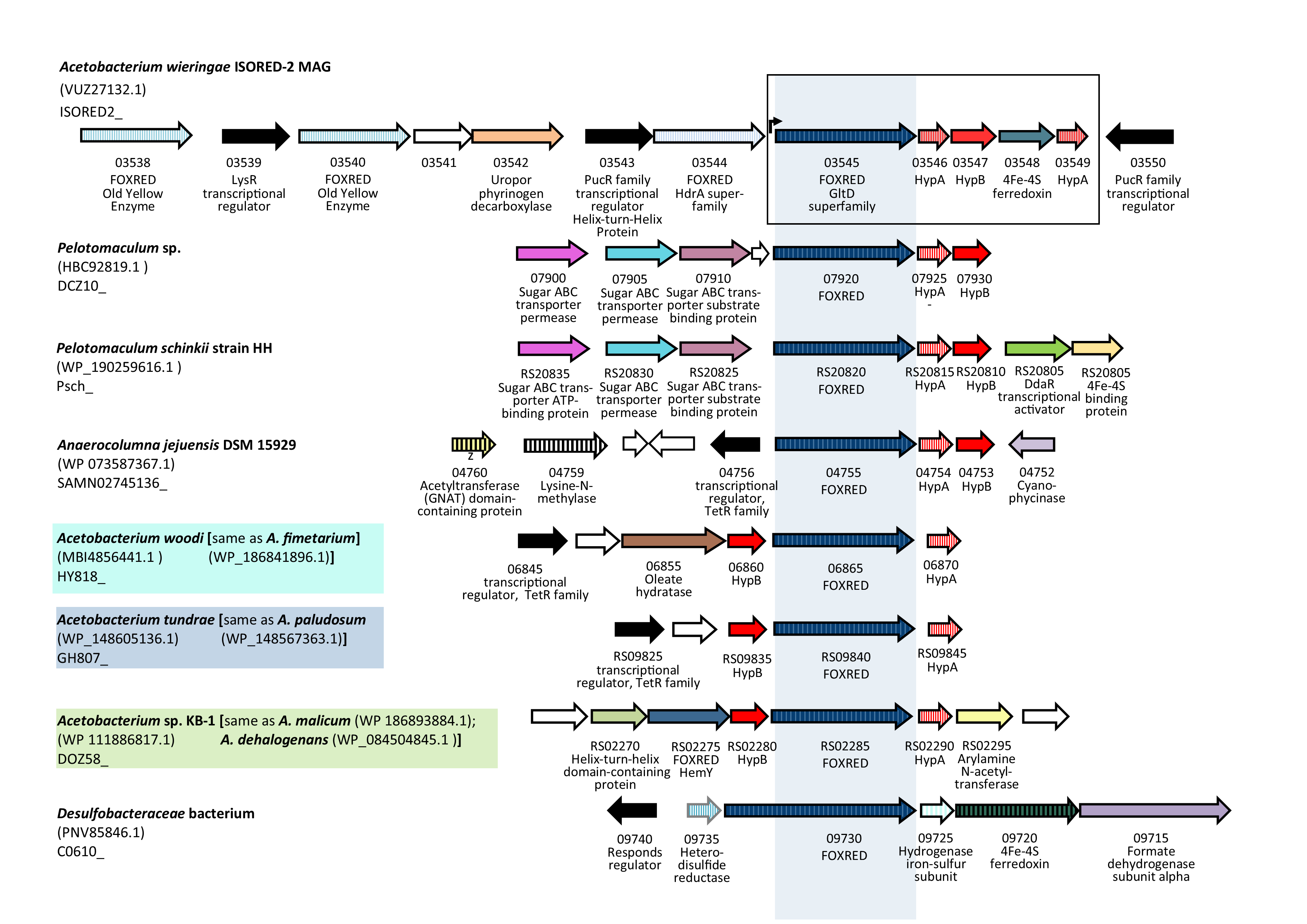

Supplement: FIG S5 [file msystems.00119-23-s0006.png]

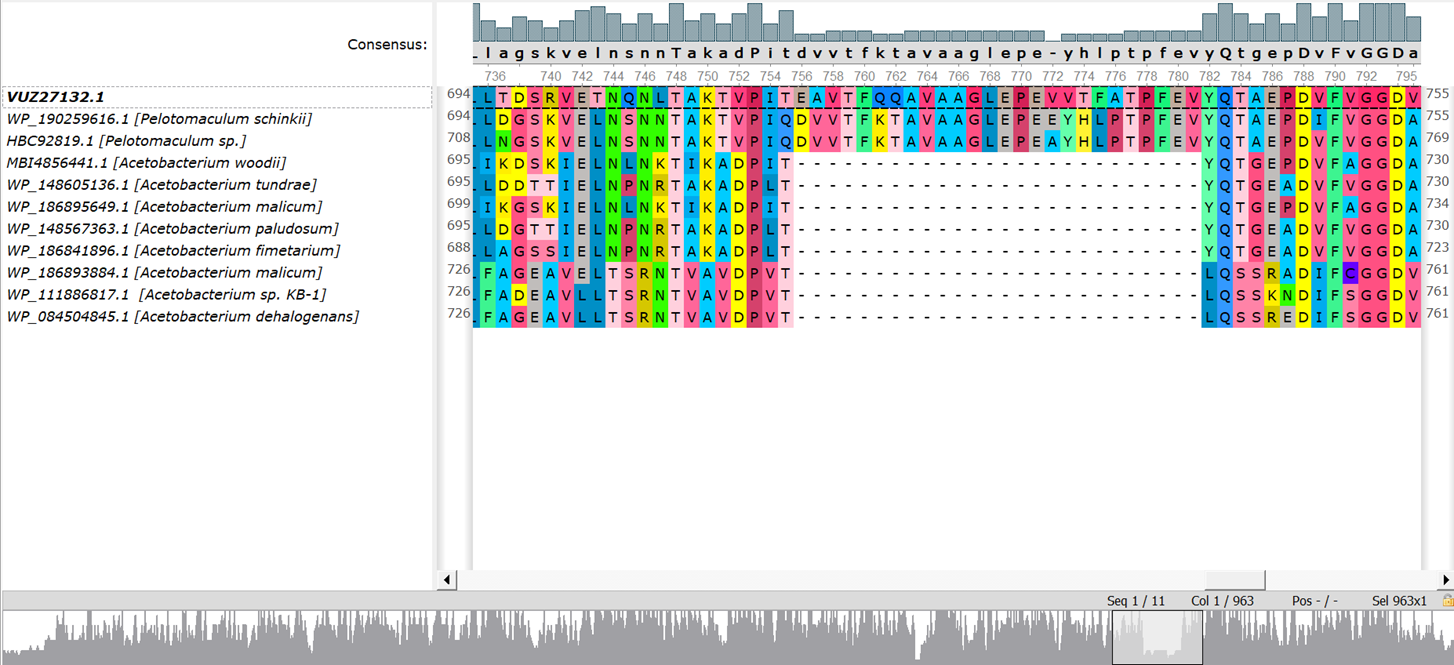

Supplement: FIG S6 [file msystems.00119-23-s0007.png]

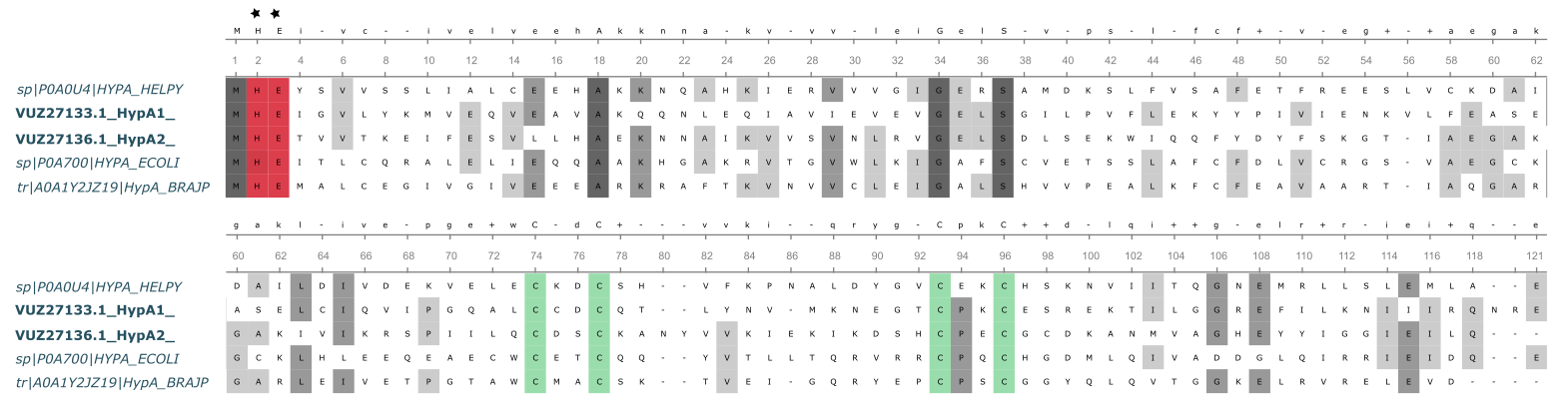

Supplement: FIG S7 [file msystems.00119-23-s0008.png]

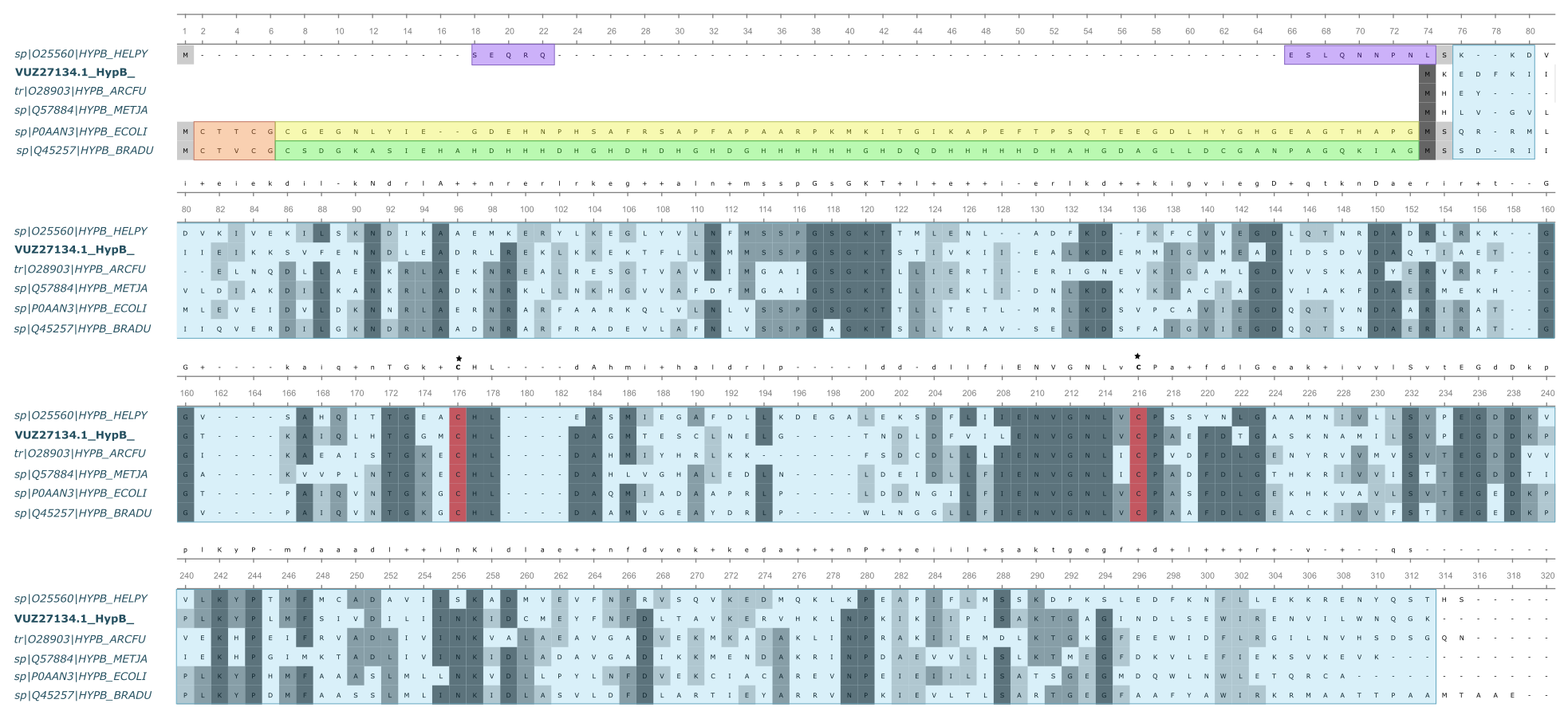

Supplement: FIG S8 [file msystems.00119-23-s0009.png]
