# Supplementary material for: Evidence for a Putative Isoprene Reductase in Acetobacterium wieringae
Source: mSystems. 2023 Mar 21;8(2):e00119-23. doi: 10.1128/msystems.00119-23 (PMC10134865; doi:10.1128/msystems.00119-23)
Supplement: TEXT S1 [file msystems.00119-23-s0001.docx]

**Supplementary Material for**

**Evidence for a putative isoprene reductase in**

***Acetobacterium wieringae***

**Miriam Kronen^1*^, Xabier Vázquez-Campos^2*^, Marc R. Wilkins^2^,
Matthew Lee^1^, Michael J Manefield^1^**

^1^UNSW Water Research Centre, School of Civil and Environmental Engineering, UNSW, Sydney, NSW 2052, Australia

^2^School of Biotechnology and Biomolecular Sciences, UNSW, Sydney, NSW 2052, Australia

# Isoprene reduction is induced in the presence of isoprene, H_2_ and HCO_3_^-^

To test whether isoprene reduction in an *Acetobacterium*-dominated (rel. abundance 16S rRNA gene amplicon sequencing 92–100%) homoacetogenic enrichment culture (1) is constitutive or inducible, cell suspensions of cells pre-grown with H_2_/HCO_3_^-^ or H_2_/HCO_3_^-^/isoprene were prepared in phosphate buffered minimal media. Cell suspensions (OD_600_ 7.5 in each flask) were incubated with H_2_, HCO_3_^-^ and isoprene and monitored for isoprene consumption and production of methylbutene, acetate and formate.

In cell suspensions containing cells pre-grown with H_2_/HCO_3_^-^ (**Figure S1A**), acetogenesis commenced immediately at 70 nmol min^-1^ for 135 min and then 24 nmol min^-1^ thereafter. A 100 min lag in isoprene reduction was observed, after which methylbutene formation commenced at 1.25 nmol min^-1^. The lag phase in isoprene reduction indicates that there is an induction process and may reflect the time required for induction of the enzymes catalysing isoprene hydrogenation. In contrast, in cell suspensions containing cells pre-grown with H_2_/HCO_3_^-^/isoprene (**Figure S1B**), both isoprene reduction and acetogenesis commenced immediately. Initial methylbutene formation and acetogenic rates were 30 nmols min^-1^ and 37 nmols min^-1^, respectively. Isoprene reduction stopped after 135 mins, at which time the acetogenic rate increased to 77 nmol min^-1^.

# Promotor prediction isoprene-regulated operon

**DNA sequence analysis.** Promoter prediction was performed using the BPROM program and operon prediction was performed with FGENESB which are available through the Softberry website ([www.softberry.com](http://www.softberry.com)).

For promoter-prediction analysis (BPROM) revealed a potential transcription start site around 44 bp upstream of the open reading frame (ORF) 1 (ISORED2_03545; putative isoprene reductase) start codon (sequence of -10 box ‘TGTTATAAT’ and sequence of -35 box ‘ATGTCA’) (**Figure S2**). Two potential transcription-factor (RNA polymerase sigma factor rpoD17 and ihf) binding sites were predicted at 58 bp and 38 bp upstream of the ORF 1 start codon. Although a strong candidate, this transcription start site has not been verified by 5’RACE or equivalent. Additionally, 4 transcription factor (TF) binding sites could be identified 13 bp upstream of the ISORED2_03545 start codon (using FIMO with the CollectTF database, output filtered by *p*-value (≤0.0001), *q*-values (≤0.05), keeping only matches in the forward sequence). They are highly suggestive binding sites for a [Fur](http://www.collectf.org/browse/view_reports_by_TF_instance/52/) (ferric uptake regulator) or [NikR](http://www.collectf.org/browse/view_reports_by_TF_instance/103/) (nickel uptake regulator) type of TF, belonging to COG0735 and COG0864 respectively.

# Genome environment of the putative isoprene-regulated operon

The putative isoprene-regulated operon is located between 69,745–75,048 bp in a 90,374 bp contig (**Figure 3B**). The first half of this contig contains mainly protein-coding genes of viral origin (exact coordinates depend on the prediction tool). The mean contig coverage and the coverage of the proviral portion are close to the values for the MAG indicating no active viral replication. The provirus (*Siphoviridae*) shows an average amino acid identity of 59.11% with the *Erysipelothrix* phage Φ1605 (2) based on CheckV, and tBLASTx of many of the viral proteins also show similar identities with several *Streptococcus* phages recently sequenced (3). This proviral region also appears in other *Acetobacterium* spp. genomes including *Acetobacterium wieringae* DSM 1911 and *Acetobacterium* sp. KB-1 (**Figure 3AC**).

The contig contains three different Ser-recombinases (integrases). Two of them, the ones adjacent to the provirus (**Figure 3**), show very high identity values with recombinases found in *A. wieringae* DSM 1911, *A.* sp. MES1, and *A.* sp. KB-1 and might be part of the provirus itself.

As the provirus and recombinases were nearly the only sequences sharing some resemblance to extant assembled *Acetobacterium* spp. genomes, the contig encoding the putative isoprene-regulated operon was carefully examined: I) by evaluating the placement of the contig in the assembly graph (possible mis-binning); and II) by mapping the reads back to the MAG to assess the possibility of misassemblies. Examination of the metagenome assembly graph in bandage clearly shows two main subassemblies in the MegaHIT assembly, corresponding to the genomes of the two MAGs recovered through binning. Visualisation in IGV provided evidence of a potential misassembly around 16,600–16,750 bp based on a severe drop in the coverage (~1% of mean coverage). Therefore, ISORED-2 was reassembled iteratively using MIRA v5rc2 in a similar way as described by (4).

After 6 iterations, the reassembled ISORED-2 showed a size increase of ~40 kbp (only contigs >2.5 kbp), but more importantly, a more polished isoprene-regulated operon contig. The polished contig dropped the first 16,660 bp (at the point of the coverage drop) but increased its total length to 178,423 bp. This contig showed a higher degree of synteny with the genome of *Acetobacterium* sp. KB-1 (**Figure 3C**), the most complete *Acetobacterium* spp. genome to date and the only one assembled in a single contig. While MIRA was able to extend the putative isoprene-regulated operon contig and provide a better overlook of the gene neighbourhood of the operon, it did it at the expense of collapsing a number of insertion sequences (IS) present around what would be the end of the original contig after the last Ser-recombinase gene, around ~73,550 and 80,500 bp. Most of this IS-rich region have coverages between 200–300x, with exception of the IS21 between 76,632-79,180 bp that has a coverage 500–750x. With most of the contig with a maximum coverage of 100–150x (only isoprene sample mapped), it is fair to assume that all, if not most, IS present here appear in tandem repeats.

# References

1. Kronen M, Lee M, Jones ZL, Manefield MJ. 2019. Reductive metabolism of the important atmospheric gas isoprene by homoacetogens. ISME J 13:1168–1182.

2. Gu J, Li YX, Xu CW, Xie XJ, Li P, Ma GX, Lei CW, Liu JX, Zhang AY. 2020. Genome sequence of multidrug-resistant *Erysipelothrix rhusiopathiae* ZJ carrying several acquired antimicrobial resistance genes. J Glob Antimicrob Resist 21:13–15.

3. Rezaei Javan R, Ramos-Sevillano E, Akter A, Brown J, Brueggemann AB. 2019. Prophages and satellite prophages are widespread in *Streptococcus* and may play a role in pneumococcal pathogenesis. Nat Commun 10:4852.

4. Lui LM, Nielsen TN, Arkin AP. 2021. A method for achieving complete microbial genomes and improving bins from metagenomics data. PLoS Comput Biol 17:1–25.
