# Supplementary material for: Evidence for a Putative Isoprene Reductase in Acetobacterium wieringae
Source: mSystems. 2023 Mar 21;8(2):e00119-23. doi: 10.1128/msystems.00119-23 (PMC10134865; doi:10.1128/msystems.00119-23)
Supplement: FIG S4 [file msystems.00119-23-s0005.pdf]

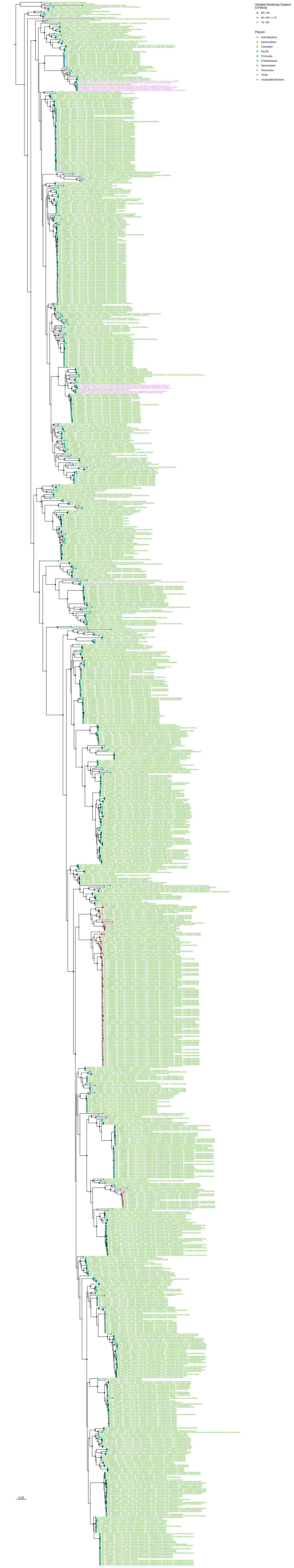

Ultrafast Bootstrap Support (UFBoot)
BP ≥ 90
90 > BP => 75
75 > BP
Phylum
Actinobacteria
Bacteroidetes
Chloroflexi
FALSE
Firmicutes
Proteobacteria
Spirochaetes
Tenericutes
TRUE
Unclassified Bacteria
0.25
